# Within-week differences in external training load demands in elite volleyball players

**DOI:** 10.1186/s13102-022-00568-1

**Published:** 2022-11-01

**Authors:** Zeki Akyildiz, Henrique de Oliveira Castro, Erhan Çene, Lorenzo Laporta, Coskun Parim, Emre Altundag, Cengiz Akarçeşme, Giovanni Guidetti, Giovanni Miale, Ana Filipa Silva, Hadi Nobari, Filipe Manuel Clemente

**Affiliations:** 1 Sports Science Department, Gazi University, Ankara, Turkey; 2 Faculdade de Educação Física, Universidade Federal de Mato Grosso, Cuiabá, Brazil; 3 Department of Statistics, Yildiz Technical University, Istanbul, Turkey; 4 Centro de Educação Física e Desportos da, Universidade Federal de Santa Maria, Santa Maria, Brazil; 5 Vakıfbank Women’s Volleyball Team, Istanbul, Turkey; 6 The Research Centre in Sports Sciences, Health Sciences and Human Development (CIDESD), 5001-801 Vila Real, Portugal; 7 Escola Superior Desporto e Lazer, Instituto Politécnico de Viana do Castelo, Rua Escola Industrial e Comercial de Nun’Álvares, 4900-347 Viana do Castelo, Portugal; 8 Department of Exercise Physiology, Faculty of Educational Sciences and Psychology, University of Mohaghegh Ardabili, 5619911367 Ardabil, Iran; 9 Faculty of Sport Sciences, University of Extremadura, 10003 Cáceres, Spain; 10 Department of Motor Performance, Faculty of Physical Education and Mountain Sports, Transilvania University of Braşov, 500068 Braşov, Romania; 11 Delegação da Covilhã, Instituto de Telecomunicações, 1049-001 Lisboa, Portugal

**Keywords:** Local positioning system, Workload, Woman players, Microcycles, Athlete tracking technology

## Abstract

**Purpose:**

The aim of this study was to analyze the within-week differences in external training intensity in different microcycles considering different playing positions in women elite volleyball players.

**Methods:**

The training and match data were collected during the 2020–2021 season, which included 10 friendly matches, 41 league matches and 11 champions league matches. The players’ position, training/match duration, training/match load, local positioning system (LPS) total distance, LPS jumps, accelerations, decelerations, high metabolic load distance (HMLD), acute and chronic (AC) mean and AC ratio calculated with the rolling average (RA) method and the exponentially weighted moving average (EWMA) method, monotony and strain values were analyzed.

**Results:**

All the variables except strain, Acc/Dec ratio and acute mean (RA) showed significant differences among distance to match days. Regarding the players’ positions, the only difference was found in the AC ratio (EWMA); in all microcycles, the middle blocker player showed workload values when compared with the left hitter, setter and libero.

**Conclusion:**

Overall, the analysis revealed that the intensity of all performance indicators, except for strain, acc/dec and acute mean load (RA), showed significant differences among distance to match day with moderate to large effect sizes. When comparing players’ positions, the middle blocker accumulated the lowest loads. There were no significant differences among other positions.

**Supplementary Information:**

The online version contains supplementary material available at 10.1186/s13102-022-00568-1.

## Background

Gaining a competitive advantage is one of the goals of training and studies focused on sports science [1, 2]. The adoption of recording and monitoring the training intensity has become an important tool in training, but if these data accumulate and there is no time to critically analyze, present and apply them, nothing will positively affect day-to-day training [3]. In parallel with this concern and technological advancements, several biological or physiological monitoring tools of the athlete are used to identify athletes’ responses to training demands, fatigue, recovery rates and injury risk [3–8].

Training intensity – a term that unites the constructs of training intensity, frequency and duration and represents the dose-response nature of the training stimulus imposed when describing how hard somebody is exercising [9] – can be considered the internal and external training intensity related to measurable aspects occurring internally or externally to the athlete [7]. Internal training intensity is characterized by physiological and psychological stress in response to training/competition intensity (reported in units derived from the product of exercise duration and RPE or HR) [8, 10, 11]. External training intensity, on the other hand, can be an indirect measure of internal intensity represented by the physical work encountered by the athlete (i.e. the stimulus imposed). It is not entirely representative but is practical and gives an approximate measure of the total amount of mechanical or locomotive stress generated by an athlete during exercise regardless of internal characteristics, which can be measured by the distances, velocity, or even as arbitrary units derived from accelerometers [11–14].

In this sense, it is important to understand the synergistic relationship between internal and external intensity when planning a training session [10, 15] and how the physical demands depend on the coach and the work prescribed in the training plan [7] to ensure that athletes are progressively and adequately stimulated so that their skills improve significantly. When this data is collected, they may be used to closely monitor physiological and psychological demands on an individual basis during training and competitions [16]. Furthermore, it can help identify athletes at injury risk or nonfunctional overreaching [3, 17–20].

In team sports, coaches prescribe training by considering the external training intensity to provoke a desired psychophysiological response [7, 21, 22]. For this purpose, advanced technology has contributed to the development of tools that show more detailed information about external intensity, quantifying (de)accelerations, distance, velocity, power and number of jumps, among other parameters, during exercise [23–25] through microelectromechanical systems such as global positioning systems (GPSs), or inertial measurement units (which include accelerometers, gyroscopes and magnetometers). Some applications in team sports are related to the use of the covered distance, speed, or acceleration/deceleration intensities in soccer [5, 12, 26–28]; relative distance covered, speed, accelerations intensities, or the relative number of impacts in rugby [29–31]; or accelerations, distance, speed [32–35] or total jump loads [16] in basketball. These variables are also applicable to volleyball, which necessitates high ability of motor performance, motion range, directions changing, and jumps [35–37]. In addition, these variables characterize the match/competition demands [16].

Although volleyball is a team sport, it is particularly different from the above-mentioned sports since it is a net game, while the others are invasion games. This means that volleyball is less dependent on horizontal displacement (e.g. running) and has a greater dependency on vertical displacement (e.g. jumping). Volleyball is an intermittent dynamic sport characterized by different movements (jumps, sprints and game actions) performed explosively with short rest intervals between points (30–60 s) and sets (3 min) [36, 37]. In addition, athletes play different roles with different types and frequencies of jumps and displacements and different intensities (i.e. a setter jumps an average of 179.9 times at a height of 41.1 cm, while a middle blocker jumps 123.3 times at 48 cm and an outside hitter jumps 141.7 times at 51.0 cm) [38]. Either way, these characteristics require players to have high levels of oxidative capacities and creatine phosphate and glycolytic energy systems [39].

Research on the external intensity training in volleyball has focused on quantifying the number and height of jumps and displacements. Lima et al. [11] verified the relationship by evaluating intra-weekly changes in elite male professional volleyball players using an inertial measurement unit (Vert® Classic, MyVert, Florida, USA) to measure the number and height of jumps as external intensity. The results revealed a strategy for reducing the external load before a match such that differences within a week would show significantly more jumps two days before the match than one day before. Lima et al. [38] aimed to assess the jump-training intensity of different playing positions (middle blockers, setters and outside hitters) in male professional volleyball players during regular competitive microcycles. The jump heights and jump moments were recorded using an inertial measurement device (Vert® Classic, MyVert, Florida, USA). No significant differences were found in the jump intensity based on the players’ positions or the day of the microcycle. However, the setter jumped significantly more often than the other players. Sanders et al. [40] quantified volleyball athlete accelerometer-based workloads and used the neuromuscular fatigue jump test to assess on-court performance throughout a competitive season for each practice and competitive game using a validated wearable microsensor device (Catapult Sports). The results indicate that low-intensity decelerations, moderate and high-intensity accelerations and low and high intensity jumps accounted for 91.7% of the differences in weekly relative power. This difference should be monitored, as excessive high-intensity jumps in practice can potentially influence on-court performance. Furthermore, only high-intensity jumps were significantly different between practices that occurred prior to winning and losing games. Hank et al. [41] evaluated the directional properties of women elite volleyball players’ movements through a 3D kinematic analysis of movement by software TEMA Bio v2.3. (Image Systems Ltd., Sweden). Middle-blockers and the libero participated the most in total rallies; forward and right movement direction and lengths from 0.7 to 2 m were predominant in the results. Therefore, the studies highlight the importance of controlling intensity to provide a necessary measure for the accurate perception of the impact of training stimuli on players and reducing the jumping training intensity on the day before the competition.

The organization, quality and quantity of a training plan determine the external training, which is defined as the physical work prescribed in the training plan [23, 42]. However, this theme still remains scarce in women’s volleyball, especially regarding intra-weekly differences considering the different athletes’ roles. Therefore, this study aimed to analyze the within-week differences in external training intensity in different microcycles considering different playing positions in women’s elite volleyball players.

## Methods

### Participants

This study included 14 top elite female club world champion volleyball league players (mean ± standard deviation (SD); age, 22 ± 0.9 years; height, 195.1 ± 7.6 cm; body mass, 71.4 ± 6.3 kg)). Volleyball players in this team participated in the competitions organized by the Turkish Volleyball Federation (TVF) and the Fédération Internationale de Volleyball (FIVB). The study was conducted in accordance with the Declaration of Helsinki. Before the study began, the players signed informed consent to participate in this study, which was approved by the Gazi University Review Board (GURB Approval Number: 2021 − 795).

### Study Design

The athlete group of this study consisted of very high-level athletes. These players were playing for a top elite women’s volleyball team competing in championships around the world and in Europe. An observational cohort study was conducted on this professional volleyball team for one season (2020/2021). In this study, both internal and external training load data were recorded during training sessions and matches. The internal training loads of the athletes were obtained by multiplying the perceived effort (RPE) degree by duration (in minutes). External loads in training sessions and competitions were recorded with LPS [KINEXON, GMBH, Precision Technologies, KINEXON ONE Munich, Germany] technology. In all training sessions and competitions, the players wore the LPS units in a specially positioned vest between the shoulder blades.

### Data

The data consisted of training and match data from the 2020–2021 season for a woman volleyball team of 14 players. During the season, 10 friendly matches, 41 league matches and 11 champions league matches were played.

The data contained players’ position, training/match duration, training/match load, LPS [KINEXON, GMBH, Precision Technologies, KINEXON ONE Munich, Germany] total distance, LPS Jumps, accelerations, decelerations, high metabolic load distance (HMLD), acute and chronic mean and AC ratio calculated with the rolling average (RA) method and the exponentially weighted moving average (EWMA) method, monotony and strain values. Firmware versions and application versions were always up-to-date when LPS data were received. The installation and calibration of the system were guided by the manufacturer’s technicians. Calibration was performed at the local measurement area during the LPS setup using a millimeter-accurate Tachymeter. This calibration was important for obtaining the exact 3D positions of all antennas. A total of 12 antennas were placed at different locations and distances of the field.

There are several studies [43–46] examining the validity and reliability of KINEXON LPS. In the study of Fleureau et al.,[44] the validity of KINEXON LPS technology was compared to the gold standard (i.e. the VICON motion capture system). According to the results, the standardized typical error of the prediction values was low (0.06), the standardized bias ranged from 0.01 to 2.85, and all Pearson coefficient values were reported to be high (> 0.90). In the study[46] examining the validity and reliability of the validity and reliability parameters of the KINEXON LPS technology, the KINEXON LPS showed a high degree of validity and reliability (typical estimation error: 1.0-6.0%; coefficient of variation: 0.7%). − 5.0% typical estimation error: 2.1–9.2%; coefficient of variation: 1.6–7.3%.

Possible positions for the players were left hitter (LH), right hitter (RH), middle blocker (MB), setter (S) and libero (L). The data also contained a variable called microcycle, which indicated the number of days until the next game, with possible values of five days before the match day (MD-5) (n = 107, number of sessions = 7), four days before the match day (MD-4) (n = 225, number of sessions = 14), three days before the match day (MD-3) (n = 436, number of sessions = 18), two days before the match day (MD-2) (n = 517, number of sessions = 28), one day before the match day (MD-1) (n = 940, number of sessions = 56), the match day (MD) (n = 811, number of sessions = 63), a match day with training on the same day (MD-T) (n = 391, number of sessions = 39) and the day after the match (MD + 1) (n = 391, number of sessions = 22).

### Statistical analysis

Before conducting the statistical analysis, LPS total distance, LPS jumps, acceleration, deceleration, HMLD and training/match load were normalized by dividing each variable into training or match duration to eliminate the effect of time. The other variables were not normalized; normalizing them would not be beneficial due to their definitions since they were defined as percentages. All the statistical analyses were conducted both with standardized and unstandardized variables.

First, the mean and standard deviation for variables were reported separately for each microcycle. Afterward, the mean values of the variables for each player in each microcycle were calculated to eliminate the different number of games that players were involved in. The Shapiro-Wilks normality test was conducted with the p-value set to 0.05 for all the variables. The results of this test indicated that the data were distributed normally. The differences between load and LPS variables among microcycles were revealed by a repeated ANOVA, and the differences between load and LPS variables among positions were revealed with an ANOVA. If any significant result was detected after the ANOVA or repeated ANOVA, Tukey’s HSD and Bonferroni’s posthoc tests were conducted for multiple comparisons of microcycles and positions, respectively. Standard error of measurement (SEM) values were calculated for each variable. As SEM values increase, the reliability between observers would decrease. More precisely, if the reliability is zero then the SEM would be equal to the standard deviation of the observed test scores and if the reliability is 1 then the SEM value would be 0. Eta squared values were also reported for the effect sizes. ${\eta }^{2}$values in the range 0-0.009 were considered insignificant effect sizes, 0.01-0.0588 were considered small effect sizes, 0.0589–0.1379 were considered medium effect sizes, and values greater than 0.1379 were large effect sizes [47]. Data visualization techniques were employed to explain the differences among microcycles and among positions. All data processing steps and analyses were conducted with the R programming language. All p-values lower than 0.05 were considered significant.

## Results

Table 1 contained the mean and standard deviation values for all the variables for all the microcycles as well as the repeated ANOVA test results. ${\eta }^{2}$ effect sizes were also given in Table 1. All the variables except for the strain, Acc/Dec ratio and acute mean (RA) showed significant differences among distance to match days. Source of difference column summarized Tukey HSD multiple comparison results for the significant pair of variables.

**Table 1:** Mean, standard deviations, repeated ANOVA and effect size results for training load metrics across different microcycles

Variables	MD-5	MD-4	MD-3	MD-2	MD-1	MD	MD-T	MD + 1	F	p	Source of Difference*	${\eta }^{2}$ Effect Size
Workload (A.U)	872.47 ± 155.77	974.16 ± 91.37	845.31 ± 73.11	890.7 ± 71.08	710.4 ± 49.45	627.48 ± 192.91	295.13 ± 41.74	846.82 ± 59.91	91.57	0.000	(MD), (MD-1) – (MD-2), (MD-3), (MD-4), (MD-5), (MD-T), (MD + 1); (MD-2) – (MD-3) – (MD-4) – (MD-T); (MD-4) – (MD-T) – (MD + 1); (MD-5) – (MD-T)	0.800 (Large)
LPS Total Distance (m)	3682.54 ± 440.43	4376.83 ± 379.72	3762.66 ± 355.15	3630.39 ± 391.26	3125.80 ± 298.58	2705.46 ± 454.54	2766.62 ± 679.97	3451.68 ± 393.96	71.93	0.000	(MD) – (MD-1) – (MD-2) – (MD-3) – (MD-4) – (MD + 1); (MD-5) – (MD), (MD-1), (MD-4); (MD-T) – (MD-2), (MD-3), (MD-4), (MD-5), (MD + 1)	0.604 (Large)
LPS Jumps (N)	100.09 ± 49.18	99.85 ± 43.28	108.91 ± 50.41	103.09 ± 45.06	88.21 ± 39.38	67.31 ± 31.84	66.41 ± 35.71	104.06 ± 48.41	17.00	0.000	(MD) – (MD-1), (MD-5); (MD), (MD-1) – (MD-2), (MD-3), (MD-4), (MD + 1); (MD-T) – (MD-2), (MD-3), (MD-4)	0.123 (Moderate)
Acceleration (m/s^− 2^)	120.01 ± 32.55	119.72 ± 30.19	124.91 ± 31.47	122.49 ± 31.55	103.59 ± 23.95	82.13 ± 29.52	83.39 ± 37.61	116.98 ± 31.98	28.16	0.000	(MD) – (MD-1), (MD-5); (MD), (MD-1) – (MD-2), (MD-3), (MD-4), (MD + 1); (MD-T) – (MD-2), (MD-3), (MD-4), (MD-5), (MD + 1); (MD-3) – (MD + 1)	0.227 (Large)
Deceleration (m/s^− 2^)	102.79 ± 31.69	103.78 ± 27.67	111.01 ± 33.01	106.53 ± 29.95	89.04 ± 23.48	68.30 ± 25.75	69.89 ± 34.03	102.87 ± 31.31	24.62	0.000	(MD) – (MD-1), (MD-5); (MD), (MD-1) – (MD-2), (MD-3), (MD-4), (MD + 1); (MD-T) – (MD-2), (MD-3), (MD-4), (MD-5), (MD + 1)	0.229 (Large)
HMLD (m)	1040.09 ± 283.25	1153.15 ± 305.38	1072.24 ± 275.94	1065.14 ± 284.45	933.29 ± 240.87	891.56 ± 250.56	942.55 ± 309.02	1004.89 ± 270.16	14.95	0.000	(MD), (MD-1) – (MD-2), (MD-3), (MD-4), (MD-5); (MD + 1) – (MD-1), (MD-2), (MD-3), (MD-4); (MD-4) – (MD-3), (MD-T)	0.084 (Moderate)
Acute Mean Load (EWMA)	814.51 ± 113.41	808.26 ± 73.89	946.62 ± 81.42	913.44 ± 82.08	867.84 ± 71.28	818.39 ± 96.08	820.87 ± 116.54	911.27 ± 78.83	22.73	0.000	(MD) – (MD-3), (MD + 1); (MD-2) – (MD), (MD-1), (MD-4), (MD-5), (MD-T); (MD-3) – (MD + 1); (MD + 1), (MD-3) – (MD-1), (MD-4), (MD-5), (MD-T);	0.256 (Large)
Chronic Mean Load (EWMA)	730.19 ± 95.16	759.41 ± 69.88	792.94 ± 73.23	795.33 ± 73.72	787.58 ± 69.02	789.96 ± 74.30	795.34 ± 84.91	793.24 ± 68.20	11.28	0.000	(MD-4) – (MD), (MD-1), (MD-2), (MD-3), (MD-T), (MD + 1); (MD-5) – (MD-2), (MD-3)	0.082 (Moderate)
AC Ratio (EWMA)	1.10 ± 0.09	1.06 ± 0.05	1.20 ± 0.00	1.14 ± 0.05	1.10 ± 0.00	1.03 ± 0.05	1.03 ± 0.08	1.14 ± 0.05	17.00	0.000	(MD) – (MD-1), (MD-2), (MD-3), (MD + 1); (MD-1), (MD-3) – (MD-2), (MD-4), (MD-T), (MD + 1); (MD-2), (MD + 1) – (MD-4), (MD-T); (MD-3) – (MD-1), (MD-5)	0.526 (Large)
Monotony (A.U)	1.46 ± 0.25	1.33 ± 0.05	1.36 ± 0.08	1.34 ± 0.08	1.43 ± 0.08	1.47 ± 0.13	1.54 ± 0.13	1.4 ± 0.09	7.30	0.000	(MD), (MD-1) – (MD-2), (MD-3), (MD-4); (MD-T) – (MD-1), (MD-2), (MD-3), (MD-4), (MD + 1)	0.245 (Large)
Strain (A.U)	9432.29 ± 2429.85	8072.37 ± 824.00	8714.29 ± 901.86	8117.93 ± 941.81	8465.03 ± 893.17	8654.19 ± 1430.68	8910.51 ± 1753.35	8582.71 ± 990.25	3.37	0.055	-	0.086 (Moderate)
Stand Workload (A.U)	7.34 ± 0.82	7.31 ± 0.54	7.19 ± 0.59	7.37 ± 0.53	6.79 ± 0.48	5.39 ± 1.20	4.58 ± 0.53	7.19 ± 0.45	64.46	0.000	(MD) – (MD-1), (MD-2), (MD-3), (MD-4), (MD-5), (MD + 1); (MD-1) – (MD-2), (MD-3), (MD-4), (MD + 1); (MD-T) – (MD-1), (MD-2), (MD-3), (MD-4), (MD-5), (MD + 1)	0.695 (Large)
Stand_LPS Total Distance (m/min)	34.34 ± 4.70	34.58 ± 2.96	34.76 ± 3.31	32.87 ± 7.69	30.23 ± 2.84	27.70 ± 4.12	44.00 ± 11.68	30.46 ± 3.37	13.64	0.000	(MD), (MD-1) – (MD-3), (MD-4), (MD-5), (MD-T); (MD + 1) – (MD-3), (MD-4), (MD-T)	0.397 (Large)
Stand_LPS Jumps (N/min)	0.94 ± 0.49	0.79 ± 0.35	1.05 ± 0.47	0.93 ± 0.43	0.86 ± 0.39	0.68 ± 0.28	1.12 ± 0.61	0.94 ± 0.43	6.65	0.005	(MD) – (MD-1), (MD + 1); (MD-3) – (MD), (MD-1), (MD-4), (MD + 1); (MD-4) – (MD-1), (MD + 1)	0.088 (Moderate)
Stand_ Acceleration (N/min)	1.11 ± 0.33	0.94 ± 0.24	1.15 ± 0.30	1.12 ± 0.43	1.01 ± 0.24	0.78 ± 0.25	1.31 ± 0.64	1.04 ± 0.28	23.26	0.006	(MD) – (MD-1), (MD-2), (MD-3), (MD-4), (MD-5), (MD-T), (MD + 1); (MD-3) – (MD-1), (MD-4), (MD + 1)	0.153 (Large)
Stand_ Deceleration (N/min)	0.94 ± 0.29	0.81 ± 0.21	1.03 ± 0.28	0.96 ± 0.38	0.86 ± 0.22	0.64 ± 0.22	1.10 ± 0.58	0.91 ± 0.28	6.51	0.007	(MD) – (MD-1), (MD-2), (MD-3), (MD-4), (MD-5), (MD + 1); (MD-3) – (MD-1), (MD-4), (MD + 1)	0.150 (Large)
Stand_ HMLD (m/min)	9.99 ± 3.04	9.15 ± 2.43	9.89 ± 2.61	9.76 ± 3.68	9.03 ± 2.36	9.36 ± 2.70	15.29 ± 5.2	8.86 ± 2.4	18.36	0.000	(MD-T) – (MD), (MD-3), (MD-4), (MD-5), (MD + 1); (MD-3) – (MD-1), (MD-4), (MD + 1)	0.293 (Large)
Accel Max (m/s^− 2^)	16.41 ± 4.91	38.23 ± 4.49	81.46 ± 5.88	101.44 ± 9.51	138.62 ± 10.62	77.06 ± 10.29	26.01 ± 4.11	65.84 ± 7.92	741.86	0.000	(MD), (MD-3) – (MD-1), (MD-2), (MD-4), (MD-5), (MD-T), (MD + 1); (MD-1) – (MD-2) – (MD-4) – (MD-5) – (MD-T) – (MD + 1)	0.964 (Large)
Decel Max (m/s^− 2^)	-2.91 ± 0.30	-2.90 ± 0.30	-2.90 ± 0.22	-2.84 ± 0.21	-2.76 ± 0.22	-2.67 ± 0.23	-2.7 ± 0.29	-2.84 ± 0.24	6.23	0.001	(MD) – (MD-3), (MD-4)	0.113 (Moderate)
Max Speed (m/s^− 1^)	17.34 ± 1.08	17.81 ± 1.09	17.11 ± 0.96	16.94 ± 0.82	16.15 ± 0.78	15.31 ± 0.92	15.53 ± 1.28	16.69 ± 1.13	26.92	0.000	(MD) – (MD-1), (MD + 1); (MD), (MD-1) – (MD-2), (MD-3), (MD-4), (MD-5); (MD-4) – (MD-2), (MD + 1); (MD-T) – (MD-2), (MD-3), (MD-4), (MD-5), (MD + 1)	0.412 (Large)
Acc/ Dec (m/s^− 2^)	-1.07 ± 0.08	-1.05 ± 0.08	-1.03 ± 0.06	-1.04 ± 0.05	-1.04 ± 0.05	-1.04 ± 0.06	-1.05 ± 0.09	-1.05 ± 0.05	0.82	0.576	-	0.036 (Small)
Accum Acce Load (A.U)	451.34 ± 83.25	495.49 ± 67.02	499.2 ± 79.1	471.85 ± 69.47	405.72 ± 53.63	371.31 ± 77.27	376.17 ± 110.55	482.31 ± 72.59	25.20	0.000	(MD), (MD-1) – (MD-2), (MD-3), (MD-4), (MD + 1); (MD-2) – (MD-3), (MD-4); (MD-5) – (MD), (MD-3); (MD-T) – (MD-2), (MD-3), (MD-4), (MD + 1)	0.299 (Large)
Acute Mean Load (RA)	820.03 ± 138.42	824.94 ± 74.73	872.91 ± 77.57	838.74 ± 78.22	829.14 ± 71.02	815.11 ± 86.38	810.48 ± 107.8	853.60 ± 75.09	2.75	0.092	-	0.048 (Small)
Chronic Mean Load (RA)	726.08 ± 97.34	780.36 ± 72.13	774.04 ± 73.83	781.11 ± 75.56	768.99 ± 69.34	788.13 ± 72.23	796.09 ± 80.82	772.1 ± 66.76	9.08	0.003	(MD-1) – (MD), (MD-T); (MD-5) – (MD), (MD-4), (MD-T)	0.067 (Moderate)
AC Ratio (RA)	1.11 ± 0.17	1.04 ± 0.05	1.12 ± 0.04	1.09 ± 0.03	1.11 ± 0.03	1.04 ± 0.05	1.05 ± 0.05	1.14 ± 0.06	4.47	0.020	(MD) – (MD-1), (MD-3), (MD + 1); (MD-4) - (MD-1), (MD-3), (MD + 1); (MD-T) – (MD-1), (MD + 1)	0.213 (Large)

MD-5: 5 Days till match day, MD-4: 4 Days till match day, MD-3: 3 Days till match, MD-2: 2 Days till match, MD-1: 1 Day till match, MD: Match Day, MD-T: Match day with training in the morning, MD + 1: The day after the match

* Examples for Source of difference column: (A) – (B) denotes significant differences between A and B; (A)-(B)-(C) denotes significant differences for all possible pairwise combinations for A, B and C. (A)-(B),(C) denotes significant differences between A and B, and also between A and C, (A),(B) – (C) denotes significant differences between A and C, and also between B and C

For the training / match workload both MD and MD-1 had significant differences with MD-2, MD-3, MD-4, MD-5, MD-T and MD + 1. The average load statistically differed for every pairwise combination between MD-2, MD-3, MD-4, MD-T. Also further statistical differences were detected between comparisons for MD-4, MD-T and MD + 1. MD-5 and MD + 1 also had significant difference in terms of workload. Total workload variable had large effect size with the value of 0.800.

For LPS total distance, all pairwise comparisons of MD, MD-1, MD-2, MD-3, MD-4 and MD + 1 showed statistically significant differences. Also LPS total distance values had significant differences between MD-5 and MD, MD-1, MD-4 and also MD-T had significant differences compared to MD-2, MD-3, MD-4, MD-5 and MD + 1. LPS Total Distance had large effect size with 0.604. LPS Jumps in MD microcycle showed significant differences compared to MD-1 and MD-5. Also MD and MD-1 showed significant differences compared to MD-2, MD-3, MD-4, MD + 1. In addition, MD-T had significant differences with MD-2, MD-3 and MD-4. LPS jumps had moderate effect size with 0.123.

Acceleration and deceleration almost showed identical differences among microcycles. Significant differences detected between MD with MD-1 and MD-5; MD-1 with MD-2, MD-3, MD-4 and MD + 1; MD-T with MD-2, MD-3, MD-4, MD-5 and MD + 1. Only for acceleration, a significant difference between MD-3 and MD + 1 was also detected. Both of the variables had large effect sizes with values 0.227 and 0.229 respectively.

For HMLD, both MD and MD-1 showed significant differences with MD-2, MD-3, MD-4 and MD-5. MD + 1 had difference compared to MD-1, MD-2, MD-3 and MD-4. Lastly MD-4 was statistically differed in terms of HMLD from MD-3 and MD-T. HMLD had moderate effect sizes with 0.084.

Acute Mean Load (EWMA) values in MD showed differences with MD-3 and MD + 1; values in MD-2 showed differences with MD, MD-1, MD-4, MD-5 and MD-T. Also differences were spotted between MD-3 and MD + 1. Both MD-3 and MD + 1 were also had significantly different Acute Mean Load (EWMA) values compared to MD-1, MD-4, MD-5 and MD-T cycles. This parameter had large effect sizes with 0.256.

Chronic Mean Load (EWMA) on the other hand showed limited number of differences in microcycles. MD-4 cycle had significant differences with MD, MD-1, MD-2, MD-3, MD-T and MD + 1 and MD-5 cycle had significant differences with MD-2 and MD-3. Chronic Mean Load (EWMA) had moderate effect size with 0.082.

MD in AC Ratio (EWMA) had difference with MD-1, MD-2, MD-3 and MD + 1. Both MD-3 and MD-2 were statistically differed from MD-2, MD-4, MD-T and MD + 1. Also both MD-2 and MD + 1 were different from MD-4 and MD-T cycle and MD-3 was different from the MD-1 and MD-5. Value of 0.526 indicated a large effect size for this variable.

For monotony, MD and MD-1 were different than MD-2, MD-3 and MD-4. Also MD-T was different from MD-1, MD-2, MD-3, MD-4 and MD + 1. Monotony had large effect size with 0.245.

For the standardized workload variable both MD, MD-1 and MD-T had significant differences with MD-2, MD-3, MD-4, and MD + 1. MD and MD-T also had significant difference with MD-1 and MD-5. Standardized workload variable had large effect size with the value of 0.695.

For standardized LPS total distance, MD and MD-1 cycles showed significant differences with MD-3, MD-4, MD-5 and MD-T. Also MD + 1 cycle showed statistically significant differences with MD-3, MD-4 and MD-T. Standardized LPS total distance had large effect size with 0.397.

Standardized LPS jumps in MD microcycle showed significant differences compared to MD-1 MD-3 and MD + 1. Also MD-3 showed significant differences compared to MD-1, MD-4, MD + 1. In addition, MD-4 cycle was statistically different from MD-1 and MD + 1. Standardized LPS jumps had moderate effect size with 0.088.

Standardized acceleration and standardized deceleration almost showed identical differences among microcycles. Significant differences detected between MD with MD-1, MD-3, MD-4, MD-5, MD + 1. MD-3 cycle also showed difference with MD-1, MD-4 and MD + 1. Only for standardized acceleration, a significant difference between MD and MD-T was also detected. Both of the variables had large effect sizes with values 0.153 and 0.150 respectively.

For standardized HMLD, MD-T cycle had difference from MD, MD-3, MD-4, MD-5 and MD + 1. Also MD-3 cycle had difference from MD-1, MD-4 and MD + 1. Standardized HMLD had large effect sizes with 0.293.

Both cycles MD and MD-3 had significant differences from MD-1, MD-2, MD-4, MD-5, MD-T and MD + 1 in acceleration max. Also MD-1, MD-2, MD-4, MD-5, MD-T and MD + 1 had all statistically significant differences pairwise. Acceleration max had large effect size with value 0.964. Deceleration max had moderate effect size with 0.113 where only MD cycle differed from MD-3 and MD-4.

Max speed showed a lot of differences among cycles. MD cycle was different than MD-1 and MD + 1; both MD and MD-1 cycles were different than MD-2, MD- MD-4, MD-5. Also MD-4 cycle was different than MD-2 and MD + 1. MD-T showed statistical differences from all cycles except MD and MD-1. MAX Speed had large effect size with the value 0.412.

Accumulated acceleration load also showed differences in pairwise comparisons. Both MD and MD-1 showed statistically significant differences from MD-2, MD-3, MD-4, MD + 1 cycles and MD-2 differed from MD-3 and MD-4 cycle. Also MD-5 cycle differed from MD and MD-3 cycle and MD-T cycle showed difference from MD-2, MD-3, MD-4 and MD + 1 cycles. This parameter had large effect size with value 0.299.

In chronic mean load (RA), MD-1 cycle showed difference from MD and MD-T cycle. Also MD-5 cycle was different than MD, MD-4 and MD-T. This variable had moderate effect sizes with value 0.067.

AC Ratio (RA) had large effect sizes with the value 0.213. For AC Ratio (RA), MD cycle differed from MD-1 and MD-3 and MD + 1. MD-4 cycle differed from MD-1, MD-3 and MD + 1. Also MD-T differed from MD-1 and MD + 1.

Table 2 and supplementary tables gave mean and standard deviation values among positions for all the data and for each microcycles separately. SEM values are also reported in Table 2. Generally speaking, all the variables have lower SEM values than most of the standard deviations. It can be observed that a certain reliability is achieved in the dataset. SEM values of the parameters: Workload (A.U) SEM: 302,34; LPS Total Distance (m) SEM:836,95; LPS Jumps (N) SEM:32,85; Acceleration (m/s^− 2^) SEM: 31,93; Deceleration (m/s^− 2^) SEM: 29,84; HMLD (m) SEM: 283,58; Acute Mean Load (EWMA) SEM: 185,59; Chronic Mean Load (EWMA) SEM: 115,9; AC Ratio (EWMA) SEM: 0,17; Monotony (A.U) SEM: 0,41; Strain (A.U) SEM: 3628,6; Stand_ Workload (A.U) SEM: 1,6; Stand_LPS Total Distance (m/min) SEM: 19,89; Stand_LPS Jumps (N/min) SEM: 0,57; Stand_Acceleration (N/min) SEM: 0,76; Stand_ Deceleration (N/min) SEM: 0,65; Stand_ HMLD (m/min) SEM: 6,96; Accel Max (m/s^− 2^) SEM: 0,37; Decel Max (m/s^− 2^) SEM: 0,44; Max Speed(m/s^− 1^) SEM: 2,03; Acc/ Dec (m/s^− 2^) SEM: 0,14; Accum Acce Load (A.U) SEM: 116,74; Acute Mean Load (RA) SEM: 192,6; Chronic Mean Load (RA) SEM: 142,55; AC Ratio (RA) SEM: 0,3.

**Table 2:** Mean, standard deviations, one way ANOVA and effect size results for training load metrics for all microcycles across different positions

Variables	LH	L	MB	RH	S	SEM	F	p	Source of Difference*	Effect Size
Workload (A.U)	762.07 ± 372.20	719.02 ± 376.35	685.38 ± 335.55	723.02 ± 372.62	715.07 ± 366.44	302.34	6.17	0.000	LH-MB	0.006 (Nonsig.)
LPS Total Distance (m)	3694.36 ± 1023.38	3065 ± 942.59	3084.05 ± 896.57	3633.41 ± 1080.27	3333.56 ± 929.41	836.95	53.55	0.000	S - LH, L, MB, RH; LH - L, MB; L-RH	0.075 (Moderate)
LPS Jumps (N)	83.24 ± 30.84	10.51 ± 8.78	108.16 ± 46.11	98.43 ± 42.42	126.57 ± 60.41	32.85	510.30	0.000	LH - L - MB - RH - S	0.379 (Large)
Acceleration (m/s^− 2^)	131.28 ± 44.44	73.3 ± 29.17	95.12 ± 35.25	138.99 ± 48.68	98.1 ± 31.04	31.93	228.80	0.000	L - LH, MB, RH, S; LH - MB, RH, S; RH - MB, S	0.258 (Large)
Deceleration (m/s^− 2^)	118.28 ± 43.47	75.62 ± 30.76	68.77 ± 26.99	123.24 ± 46.14	88.25 ± 29.37	29.84	262.70	0.000	LH, RH - L, MB, S; L - MB, S; S-MB	0.285 (Large)
HMLD (m)	1213.35 ± 337.25	641.51 ± 255.37	935.19 ± 286.22	1273.33 ± 423.5	786.38 ± 253.68	283.58	329.40	0.000	LH - L - MB - RH - S	0.333 (Large)
Acute Mean Load (EWMA)	917.85 ± 205.34	868.36 ± 206.31	813.7 ± 190.49	877.99 ± 221.46	853.44 ± 201.66	185.59	36.63	0.000	LH - L, MB, RH, S; MB - L, RH, S	0.037 (Small)
Chronic Mean Load (EWMA)	834.09 ± 128.5	792.14 ± 128.68	738.58 ± 124.3	803.01 ± 147.02	771.57 ± 129.85	115.90	76.97	0.000	LH - L, MB, RH, S; MB - L, RH, S, RH - S	0.075 (Moderate)
AC Ratio (EWMA)	1.10 ± 0.18	1.09 ± 0.19	1.10 ± 0.18	1.09 ± 0.18	1.11 ± 0.19	0.17	0.85	0.493	-	0.001 (Nonsig.)
Monotony (A.U)	1.41 ± 0.39	1.35 ± 0.38	1.47 ± 0.51	1.41 ± 0.36	1.39 ± 0.38	0.41	9.12	0.000	LH - L, MB, RH; MB - L, RH, S	0.009 (Nonsig.)
Strain (A.U)	9008.67 ± 3822.77	8123.03 ± 3612.12	8348.64 ± 3939.25	8655.2 ± 3655.27	8230.9 ± 3491.03	3628.6	7.72	0.000	LH - L, MB, S	0.008 (Nonsig.)
Stand_ Workload (A.U)	6.82 ± 2.03	6.31 ± 2.14	6.24 ± 1.77	6.51 ± 1.99	6.30 ± 2.01	1.60	14.19	0.000	LH - L, MB, RH, S	0.015 (Small)
Stand_LPS Total Distance (m/min)	34.53 ± 11.73	28.12 ± 10.87	30.04 ± 11.62	36.69 ± 44.95	31.26 ± 11.06	19.89	13.50	0.000	LH - L, MB; RH - L, MB, S	0.020 (Small)
Stand_LPS Jumps (N/min)	0.83 ± 0.37	0.11 ± 0.10	1.08 ± 0.52	1.03 ± 1.16	1.23 ± 0.63	0.57	222.20	0.000	RH, MB - L, LH, S; L - LH, S; LH - S	0.210 (Large)
Stand_Acceleration (N/min)	1.22 ± 0.49	0.67 ± 0.34	0.91 ± 0.41	1.39 ± 1.77	0.91 ± 0.34	0.76	58.25	0.000	MB, S - LH, L, RH; LH - L, RH; L - RH	0.081 (Moderate)
Stand_ Deceleration (N/min)	1.09 ± 0.47	0.68 ± 0.35	0.66 ± 0.30	1.22 ± 1.50	0.82 ± 0.32	0.65	71.05	0.000	L, MB - LH, RH, S; LH - RH, S; LH - S	0.097 (Moderate)
Stand_ HMLD (m/min)	11.49 ± 4.41	5.95 ± 2.97	9.22 ± 4.02	12.98 ± 16.09	7.51 ± 3.40	6.96	68.10	0.000	LH - L - MB - RH - S	0.094 (Moderate)
Accel Max (m/s^− 2^)	2.92 ± 0.38	3.09 ± 0.38	2.74 ± 0.45	2.84 ± 0.41	2.99 ± 0.38	0.37	58.00	0.000	LH - L - MB - RH - S	0.081 (Moderate)
Decel Max (m/s^− 2^)	-2.84 ± 0.44	-3.14 ± 0.44	-2.63 ± 0.51	-2.66 ± 0.41	-2.94 ± 0.49	0.44	95.19	0.000	RH, MB - L, LH, S; L - LH, S; LH - S	0.126 (Moderate)
Max Speed(m/s^− 1^)	16.88 ± 2.32	17.11 ± 2.25	15.89 ± 2.14	16.31 ± 2.49	16.87 ± 2.00	2.03	28.78	0.000	LH, L, S - MB, RH; RH-MB	0.042 (Small)
Acc/ Dec (m/s^− 2^)	-1.04 ± 0.14	-0.99 ± 0.12	-1.06 ± 0.17	-1.08 ± 0.13	-1.03 ± 0.14	0.14	19.51	0.000	S, LH - L, MB, RH; L - MB, RH	0.029 (Small)
Accum Acce Load (A.U)	482.59 ± 134.34	440.81 ± 125.17	370.58 ± 106.94	475.17 ± 141.62	478.77 ± 175.45	116.74	82.53	0.000	LH, RH, S - L, MB; L - MB	0.111 (Moderate)
Acute Mean Load (RA)	880.48 ± 207.36	833.31 ± 207.27	782.27 ± 190.56	843.77 ± 220.97	819.24 ± 201.54	192.60	32.47	0.000	L, RH, S - LH, MB; LH - MB	0.033 (Small)
Chronic Mean Load (RA)	824.3 ± 157.6	782.06 ± 148.97	728.45 ± 141.82	793.02 ± 165.61	762.41 ± 159.78	142.55	55.68	0.000	L, RH, S - LH, MB; MB - LH, S - RH	0.055 (Small)
AC Ratio (RA)	1.09 ± 0.30	1.09 ± 0.31	1.09 ± 0.29	1.08 ± 0.28	1.10 ± 0.32	0.30	0.518	0.722	-	0.001 (Nonsig.)

LH: Left Hitter, RH: Right Hitter, MB: Middle Blocker, S: Setter and L: Libero, SEM: Standard Error of Measurement

* Examples for Source of difference column: (A) – (B) denotes significant differences between A and B; (A)-(B)-(C) denotes significant differences for all possible pairwise combinations for A, B and C. (A)-(B),(C) denotes significant differences between A and B, and also between A and C, (A),(B) – (C) denotes significant differences between A and C, and also between B and C

One way ANOVA with Bonferroni multiple comparison results and ${\eta }^{2}$ effect sizes were also reported in Table 2 and in supplementary tables.

Workload variable showed statistically significant differences between LH and MB in all cycles and in cycles MD-1, MD-2, MD-3, MD-4 and MD-5. Also in MD-4 there was difference between MB and S and in MD-5 there was difference between MB and L. In all the cases, players in the MB position had the lowest workload value.

In LPS Total distance variable, settlers discriminated from all other positions for all cycles together. Also LH separated from L and MB and L was separated from RH. Looking at each cycle individually gave different results though. Both LH and RH were separated from L and MB positions in MD + 1, MD, MD-1 and MD-2 and MD-3 cycles. MB and S position also showed difference for MD-5 cycle.

LPS jumps had large effect size values for all positional comparisons. For all cycles all positions showed statistically significant differences from each other. In MD + 1, all pairwise comparisons were significant except between MB and RH. In MD-T, L was separated from all other positions and RH was separated from S. In MD, L and S was separated from LH, MB and RH and also L was different than S position. In MD-2, all pairwise comparisons among positons were significant. In MD-3, all pairwise comparisons were significant except between MB and RH. In MD-4, LH was statistically significant from MB and S and L was discriminated from all other positions. Also there was difference between positions RH and S. In MD-5, L was separated from all other positions and also there was difference between S and LH, and S and MB.

In acceleration for all cycles, L and LH were separated from other remaining positions and also position L differed from LH. RHs also showed statistically significant differences compared to MB and S positions. Looking deeper for each microcyle stated LH and RH showed statistically significant differences from MB and L in all microcycles. Also LH and RH differed from S in all microcycles except MD-T. Also L and MB showed statistical differences in all microcycles except MD-T, MD and MD-5.

In deceleration for all cycles, LH and RH were separated from other remaining positions and also position L differed from MB and S. Lastly, S differed from MB. Looking into microcycles, LH and RH were separated from L, MB and S in MD + 1, MD-1, MD-2, MD-3, MD-4 and MD-5. LH and RH were different from MB in MD-T and different from MB and L in MD. S and MB positions showed differences in MD + 1, MD, MD-1, MD-2 and MD-3.

In HMLD all pairwise comparisons among positions showed statistically significant differences in all cycles together and in MD-1. For the remaining microcycles, LH and RH showed differences from L, MB and S in MD + 1, MD, MD-2, MD-3, MD-4 and MD-5. LH and RH showed differences from L and S in MD-T. Also MB and L showed differences in all cycles except MD-T.

In acute mean load (EWMA) LH and MB positions differed from L, RH and S, and also LH and MB differed from each other in all cycles together. Investigating each cycle individually gave insight that MB differed from either LH or both LH and RH in each cycle. Also MB differed from L in MD-1, MD-2 and MD-3. No significant difference was detected among positions in MD-5.

In chronic mean load (EWMA) LH and MB positions differed from L, RH and S, and also LH and MB differed from each other in all cycles together. No significant difference was detected among positions in MD-5 and both LH and RH were statistically differed from MB in all cycles except MD-5 and MD-4. In MD-4 only LH and MB showed statistically significant differences. Also LH and S differed in MD + 1, MD-T, MD, MD-1 and MD-2.

AC Ratio (EWMA) did not show any statistically significant differences among positions in any of the microcycles.

Monotony did not show any statistically significant differences among positions in most of the microcycles. The exceptions were LH differed from L, MB and RH in all data together and MB differed from L, RH and S in all the data. Also MB and L differed in MD and MB and S differed in MD-2.

Strain did not show any statistically significant differences among positions in most of the microcycles. The exceptions are LH differed from L, MB and S in all data together and S and LH differed in MD.

In standardized workload no difference was found in cycles MD and MD-3. LH showed statistical difference with all other position in the all data together. In MD + 1, MB showed difference with LH and RH. MB showed difference with RH and S in MD-T and LH also differed from S in MD-T. LH differed from L, MB and S cycles and RH differed from MB in MD-1 cycle. Both LH and RH differed from MB in MD-2 and LH also differed from L in the same cycle. LH differed from L and MB in MD-4 and MB differed from S in the same cycle. MB differed from LH and L in MD-5.

In standardized LPS total distance, no difference was found among positions in MD-T, MD-4 and MD-5. LH showed difference from L and MB, and RH showed difference from L, MB and S in all the data together. In MD + 1, LH showed difference with L and MB where in MD and in MD-1 both LH and RH showed difference with L and MB. In MD-1 also L and S differed. In MD-2, RH and MB differed and in MD-3, MB differed from LH, L and RH.

In standardized LPS jumps for all the data RH and MB differed from the positions L, LH and S and L, LH and S differed from each other. In MD + 1, S and MB differed from L and LH; RH differed from L and S; and L differed from LH. In MD-T, L differed from all other positions and LH differed from S. In MD, L and S differed from all other positions and L differed from S. In MD-1, all positions differed from each other. In MD-2, LH and L differed from MB and RH and L differed from LH and S. In MD-3, L differed from LH and in MD-4, L differed from other positions and LH diverged from MB and S. In MD-5, L differed from all other positions and LH differed from S.

In standardized acceleration different cycles gave different results. RH and LH differed from L, MB and S in MD + 1, MD, MD-1, MD-3 and MD-4. Also, L diverged from MB in MD + 1, LH differed from both L and MB in MD-T, S differed from L and MB in MD and L differed from MB and S in MD-1. In MD-2, RH differed from all other positions and L differed from MB in MD-3. In MD-4, L contrasted from MB and S and in MD-5, LH differed from L, and RH differed from L, MB and S.

In standardized deceleration, RH and LH differed from L, MB and S in MD + 1, MD-1, MD-3 and MD-4. Also RH and LH contrasted from MB in MD-T, diverged from both MB and L in MD and MD-5. S differed from L and MB in MD and in MD-1. RH differed from other positions in MD-2 and MB diverged from LH in the same cycle. Also in MD-5 RH differed from S.

In standardized HMLD, RH and LH differed from L, MB and S in MD + 1, MD, MD-1, MD-3 and MD-4. MB contrasted from L and S in MD + 1 and in MD-3; and L diverged from MB and S in MD and in MD-1. L differed from both LH and RH in MD-T and in MD-5. Also MB differed from S in MD-1, RH diverged from L, MB and S in MD-2, L differed from MB and MD-4 and RH differed from S in MD-5.

In acceleration max, no significant difference was found in MD-T and MD-5 and MB position seemed to be the source of difference in each cycle. MB showed differences with LH, L, R and S in MD + 1 and MD-1 and showed differences with LH, L and S in MD, MD-2 and MD-4, and showed differences with L and LH in MD-3. Other than that, S showed difference in LH and RH in MD + 1, L showed difference with LH and RH in MD-1, and showed difference with LH, RH and S in MD-2 and in MD-3. In MD-3, LH showed difference with RH and RH showed difference with S.

In deceleration max, RH and MB showed difference with L, LH and S, and LH showed difference with L and S in MD + 1. S separated from MB in MD-T and MB differentiated from LH, L and S, and L differed from RH in MD. L diverged from all other positions in MD-1, MB differed from LH, and S differed from MB and RH in MD-1. L differed from LH, MB and RH, and S differed from L, MB and RH in MD-2. L differed from all other positions, LH differed from MB and RH, and RH differed from S in MD-3. MB and RH differed from LH, L and S, and L differed from LH in MD-4. L diverged from LH, MB and RH from MD-5.

For max speed, MB and RH diverged from LH, L and S, and RH differed from MB for all the data and there was no significant difference in MD-T cycle. MB differed from LH, L, RH and S in MD + 1, contrasted from LH and S in MD, differed from LH, L and S in MD-1, differed LH and L in MD-2 and in MD-3, differed from LH in MD-4 and differed from L and RH in MD-5. Other than that, RH differed from LH in MD + 1, differed from L in MD-1 and diverged from LH and L in MD-3.

For Acc/Dec no significant difference was found in MD-T, MD-3 and MD-5 cycles. S and LH differed from L, MB and RH, and L differed from MB and RH for all the data. L differed from RH in MD + 1, MD-2 and MD-4, disagreed from L and LH in MD-1. Other than that, MB differed from LH, L and S in MD, L differed from MB in MD-1, differed from LH in MD-2 and differed from LH and MB in MD-4.

For accumulated acceleration load no significant difference was detected in MD-T cycle and L and MB differed from LH, RH and S, and L differed from MB for all the data. MB differed from LH, L, RH and S in MD + 1, MD-1, MD-2 and MD-3, differed from LH, RH and S in MD, MD-4 and MD-5. Other than that, L differed from RH in MD + 1, differed from LH in MD and differed from both RH and S in MD-5.

In acute mean load (RA), L, RH and S separated from LH and MB, and LH differed from MB in all the data and there was no significant difference among positions in MD-4 and MD-5. MB differed from LH in MD + 1, MD-T, MD, MD-1, MD-2 and MD-3. LH differed from S in MD, MB differed from L and RH, and S differed from LH in MD-1.

In chronic mean load (RA) no significant difference was found in MD-5 and L, RH and S differed from LH and MB, and MB diverged from LH, S and RH for all the data. MB differed from L, LH and RH in MD and MD-1, contrasted from RH and LH in MD-2 and MD-T, and differed from LH in MD + 1, MD-3 and MD-4. Also LH differed than S in MD-T, MD-1, MD-2 and differed from L and S in MD.

In AC ratio (RA), no significant difference was detected in any of the cycles.

**Fig. 1:**
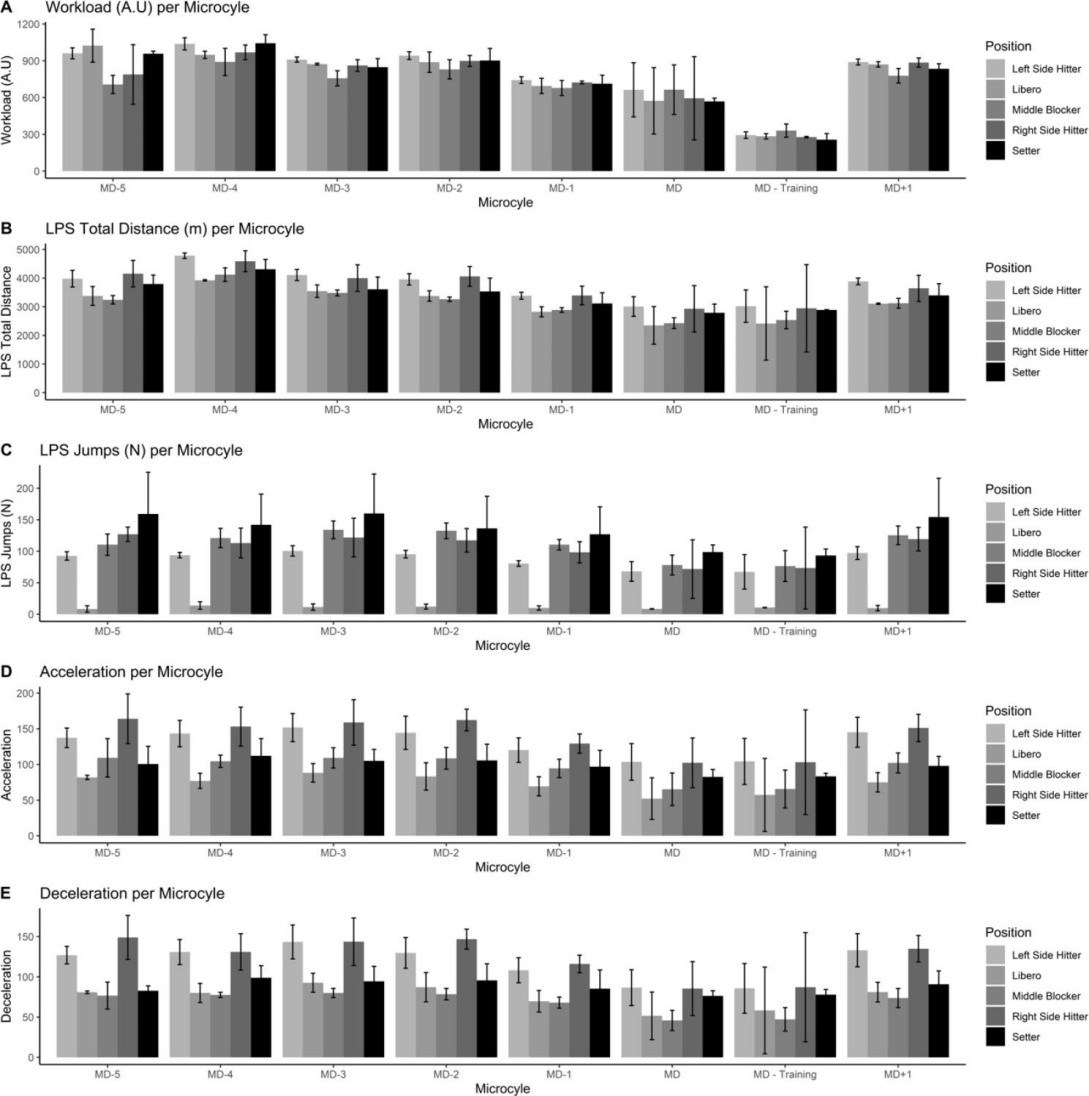
Plots of the training load metrics across different microcycles and positions [from 1 to 5 variables]

**Fig. 2:**
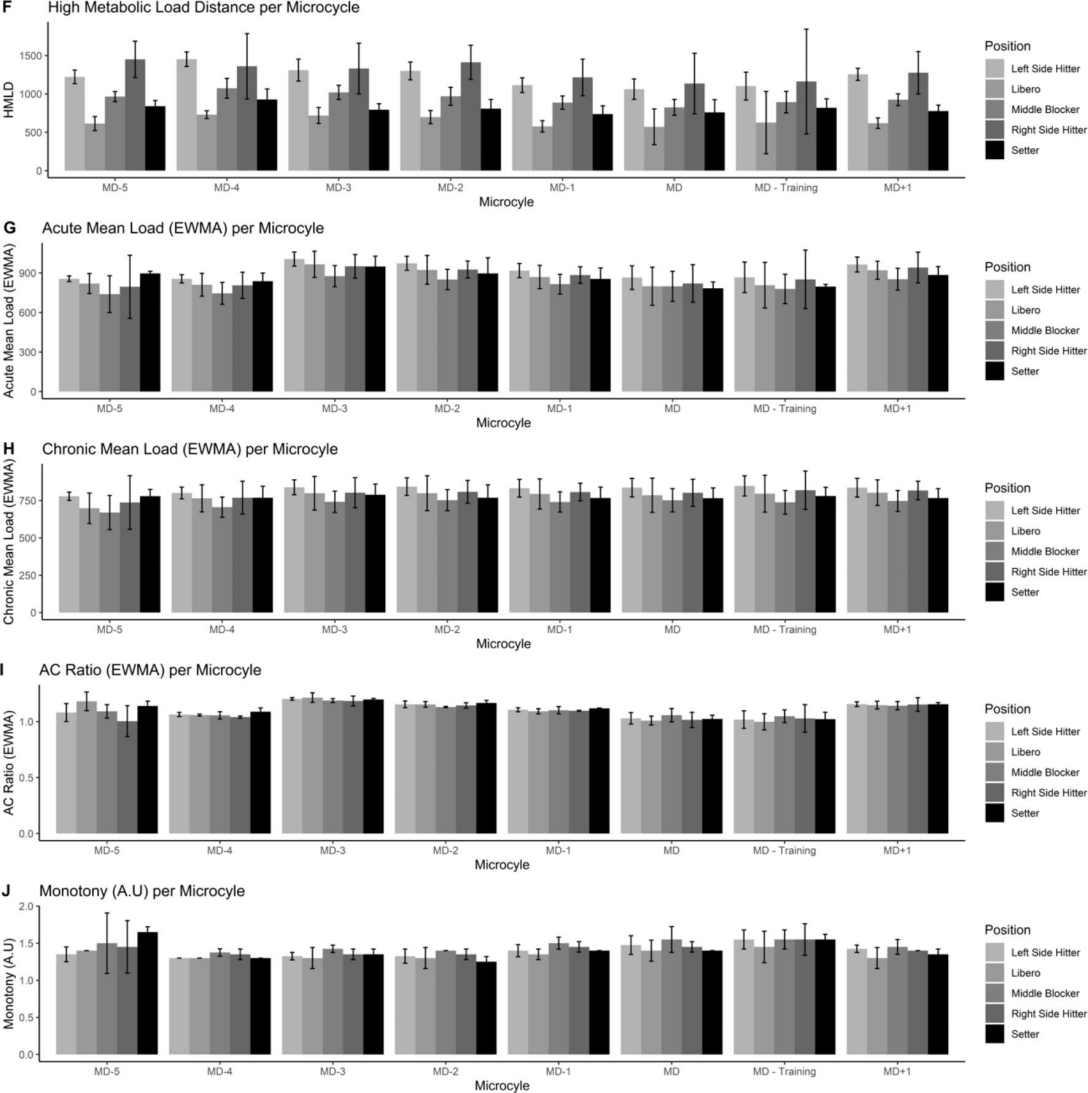
Plots of the training load metrics across different microcycles and positions [from 6 to 10 variables]

**Fig. 3:**
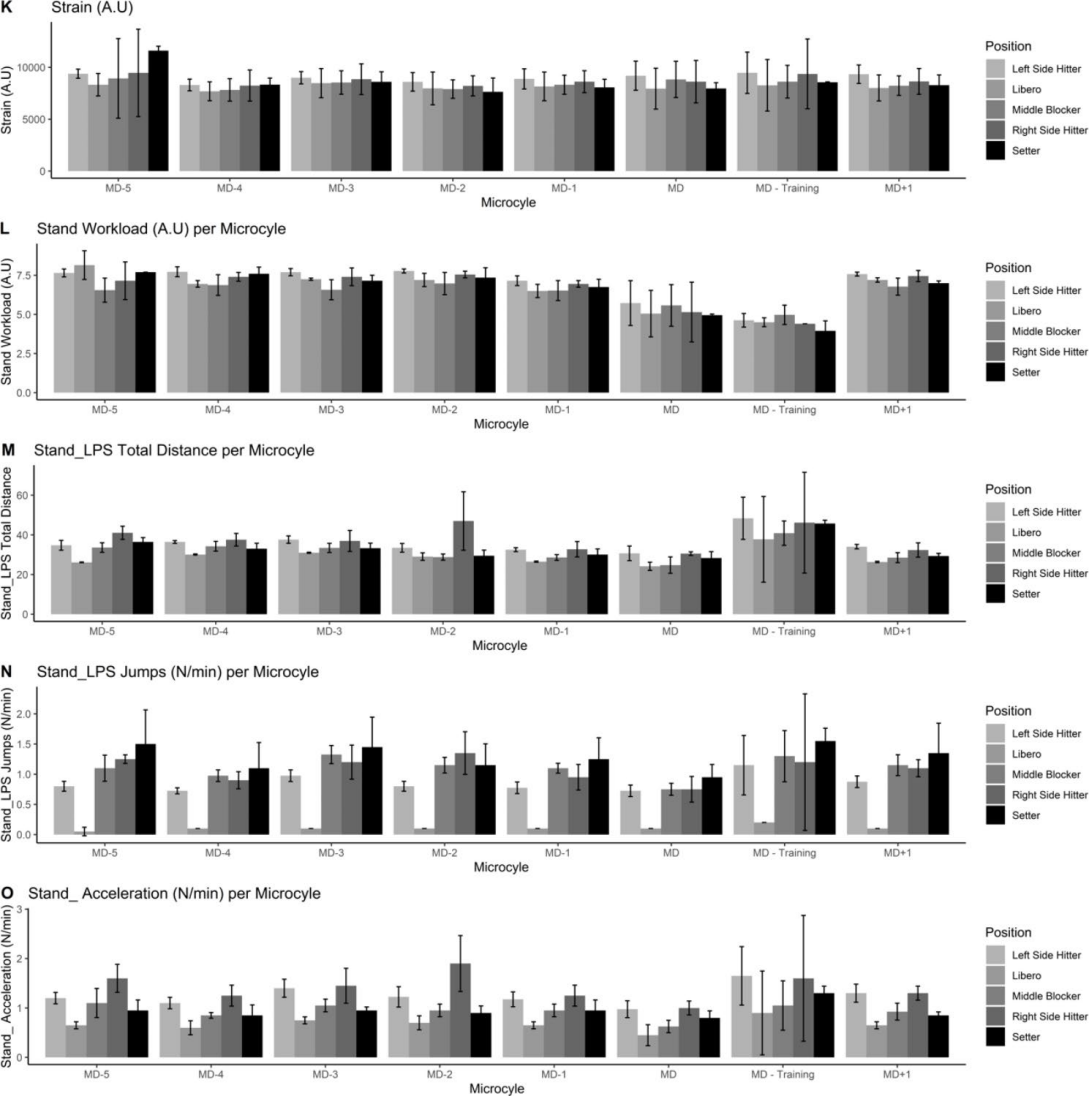
Plots of the training load metrics across different microcycles and positions [from 11 to 15 variables]

**Fig. 4:**
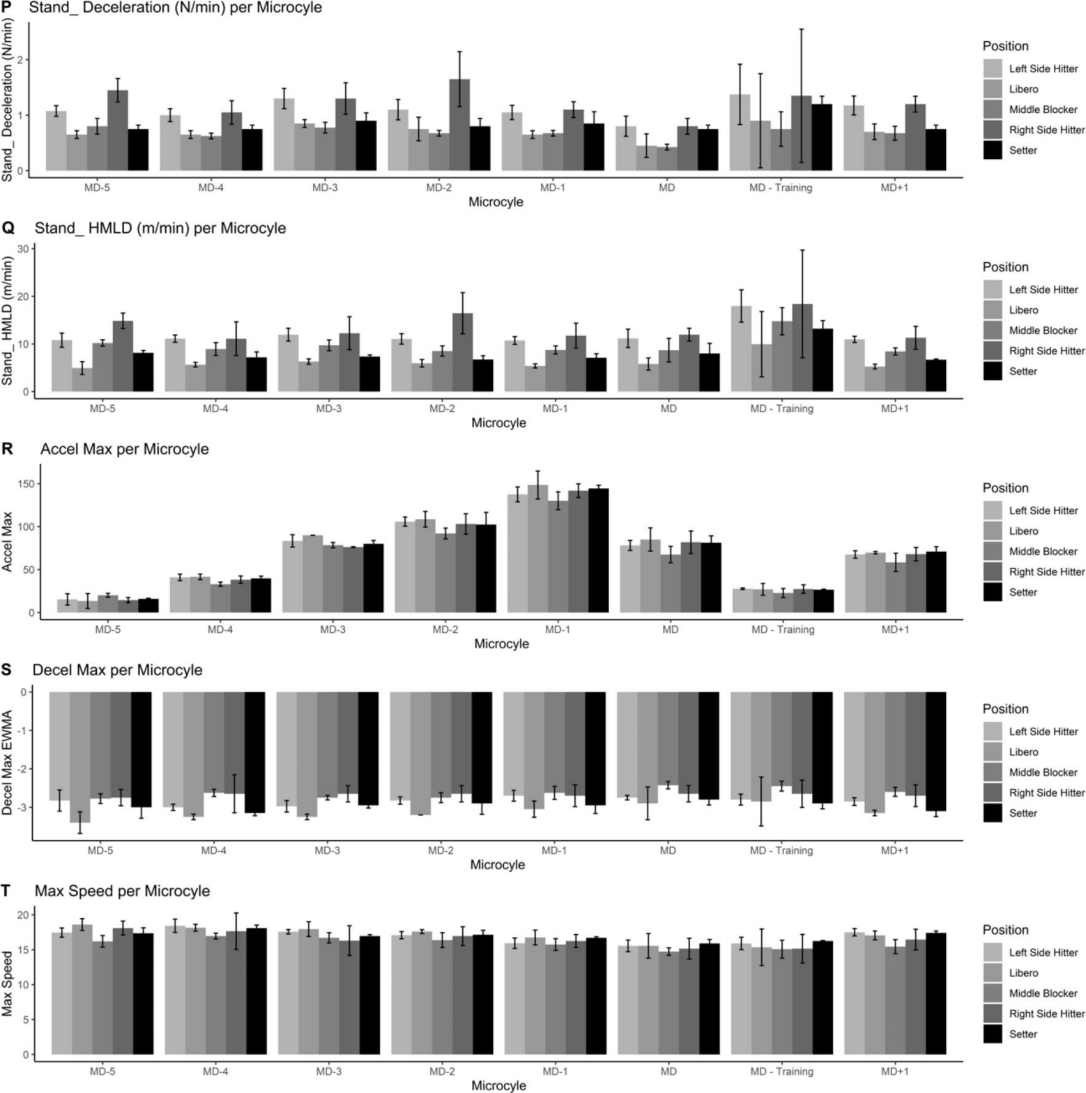
Plots of the training load metrics across different microcycles and positions [from 16 to 20 variables]

**Fig. 5:**
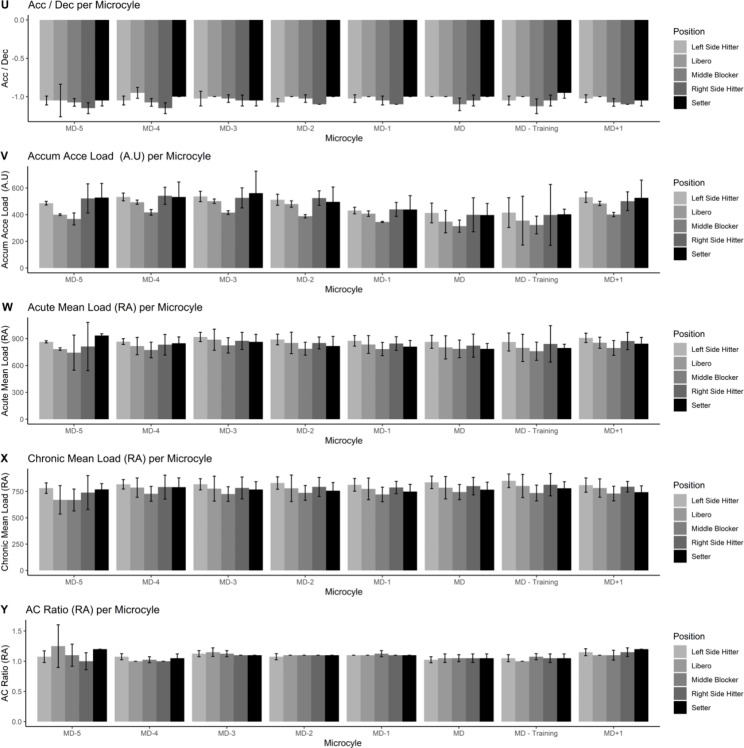
Plots of the training load metrics across different microcycles and positions [from 21 to 25 variables]

Figures 1, 2, 3, 4 and 5 also visualized the differences for each variable among position and every microcycle. Overall, intensity of the performance indicators decreased as less day remains until the match and significant differences with moderate to large effect sizes were detected (Table 1).

## Discussion

The purpose of this study was to analyze the within-week differences in external training intensity in different microcycles among women’s elite volleyball players while considering different playing positions. This study reinforces the importance of using technology to monitor athletes during training sessions and matches to improve performance and reduce the risk of injury [48] by helping coaches design accurate and specific training programs. Provides an initial understanding of monitoring the external intensity variables of elite female volleyball athletes over seven microcycles of training and one match (five days before the match: MD-5; match day: MD; match day with training in the morning: MD-T; and one day after the match: MD + 1). The results indicate significant differences in the variables representing the intensity of performance when the total sample was evaluated and when different playing positions were compared throughout the microcycles of the training and match week.

### General analysis of training intensity in the different microcycles

Overall, the analysis revealed that the intensities of all performance indicators, except for strain, acc/dec and acute mean load (RA), showed significant differences (with moderate to large effect sizes) depending on the distance to the match day.

HMLD was significantly different on MD and MD-1 when compared to MD-2, MD-3, MD-4 and MD-5; specifically, it was smaller closer to match day. Acute mean load (EWMA) values showed a reduction when comparing MD with MD-3 and MD + 1. However, when we analyzed MD-3, MD-2 and MD + 1, the acute mean load (EWMA) was larger than on MD. On the other hand, the chronic mean load (EWMA) MD-4 cycle was smaller than MD, MD-1, MD-2, MD-3, MD-T and MD + 1. In addition, the acute mean load (EWMA) was smaller on MD-5 than MD-2 and MD-3. The AC ratio (EWMA) was smaller on MD than MD-1, MD-2, MD-3 and MD + 1. For monotony, MD and MD-1 had higher values than MD-2, MD-3 and MD-4; also, this value was higher on MD-T than MD-1, MD-2, MD-3, MD-4 and MD + 1. Debien et al. [37] conducted a study on male elite volleyball athletes for one season and reported moderate correlations between week training load and strain, as well as a small correlation between total quality recovery (TQR) scores and monotony across weeks. The workload variable was significantly smaller on MD and MD-1 than MD-2, MD-3, MD-4, MD-5, MD-T and MD + 1. Our results are similar to those presented by Kupperman et al. [49], who studied collegiate women’s volleyball players and reported higher workload demand during training sessions than matches by using an accelerometer. Similar results were presented in our study based on other indicators.

For the LPS total distance and LPS jump variables, significant differences were detected between all comparisons. These values were smaller on MD and MD-T when compared to the other microcycles. Jump load is one variable that has been extensively studied for analyzing and controlling external intensity in volleyball. Our results demonstrated a decrease in the LPS jump when approaching MD. In a recent study on collegiate women’s volleyball players by Taylor et al. [50], the external load analysis (jump count – JC, jump height – JH, maximal jump performance - JH5 and jump load – JL) was performed during the season and revealed no differences in overall JC or JL between training sessions and matches. The authors explained that players who played more than 90% of sets during a match were exposed to higher loads during the match than during training. However, different results were presented by Taylor et al. [51], who reported higher jump loads in training sessions than during matches. Similar results were demonstrated in the present study, as a decrease in jump load (here represented by the total number of jumps) was observed throughout the week when approaching the match day and during the match itself. Similar findings were demonstrated in the study of Lima et al. [11] on elite men’s volleyball players; they reported significantly more jumps on MD-2 than on MD-1. It is important to highlight that the number of jumps (according to LPS jump data), which was used in the present study, is just one of the parameters used to monitor jump load in volleyball. Other parameters, including jump height and maximal jump performance [38, 39], can also be used. Mroczek et al. [52] showed the relevance of the LPS total distance analysis in volleyball. The authors demonstrated that athletes covered 1.221 ± 327 m in a three-set match and 1.757 ± 462 m in a four-set match [52] (i.e. long distances are covered in a volleyball match, and it is necessary to evaluate and control this variable).

Our results also revealed differences in other performance indicators. Acceleration presented the following small significant differences: MD with MD-1 and MD-5; MD-1 with MD-2, MD-3, MD-4 and MD + 1; MD-T with MD-2, MD-3, MD-4, MD-5 and MD + 1; and MD-3 with MD + 1. The deceleration data showed similar results. In line with these findings, Harper et al. [4] stated that elite players need to be exposed to the demands during training sessions to ensure they are prepared for high-intensity acceleration and deceleration during the match and competitive phase. A recent study by Garcia et al. [53] on male soccer players showed that regular weeks presented greater acute, monotony, strain indices and workload than congested weeks, independent of the level of participation during matches. These results approximate ours in part. Show that workload and monotony are higher on training days than match days; however, no such differences occurred for acute and strain indices. This difference may have occurred due to the specificity of the training and modalities since soccer is an invasion sport involving contact between opponents.

### Training intensity in the different microcycles according to the playing position

In volleyball, the different tasks of athletes on the court can influence the individual demands of each athlete [38, 49, 54, 55]. Thus, to understand this difference, all analyses of training intensity were performed by separating athletes by playing position. This analysis indicates differences on an individual player level. Of all the performance indicators analyzed, only the AC ratio (EWMA) showed no differences between playing positions. Our results indicated that the LH and MB showed a difference in load. In addition, our results showed differences in the workload values in all microcycles; they were lower in the MB position than in the LH, S and L positions.

Regarding LPS total distance, the S traveled greater distances than all other positions for all microcycles combined. In addition, the LH and RH were different from the L, and the LH was different from the MB. The dynamics of the volleyball game using only one S on the court could explain the greater distance covered by athletes in this position.

In the LPS jumps analysis, for all cycles, all positions show statistically significant differences from each other. In general, athletes jump more often in training microcycles than on the match day, with the highest occurrence between S and MB. These results are in agreement with others presented in the literature. Probably, these athletes jump more often than others, whether to perform a touch, block or attack, according to the specific functions of each action.

Evaluating the external training intensity in Italian elite female volleyball athletes, Ungureanu et al. [55] showed that S and MB performed the highest occurrence of jumps in training compared to hitters and opposites. In another study on women’s collegiate volleyball players in different playing positions using an accelerometer, Kupperman et al. [49] demonstrated that the setter accumulated the most jumps of any position in matches and performed three times as many medium jumps in training sessions than in matches. Herring and Fukuda [54] showed that outside hitters had the highest mean jump height, followed by MB and right-side hitters. On the other hand, middle blockers had higher jump numbers than outside hitters and right-side hitters. In line with these findings, Lima et al. [38] observed elite male volleyball players to assess the external jump-training load of different playing positions during regular competitive microcycles. Their results indicated differences in the total number, intensity and frequency of jumps for the different playing positions per training session. Specifically, the setters did a higher amount of jumps than the other playing positions evaluated. No differences were found in the intensity, and significantly fewer jumps were recorded on the day before the competition.

Furthermore, our results showed differences between playing positions in terms of accelerations, decelerations, HMLD, acute and chronic mean load (EWMA), monotony and strain in different microcycles. These results corroborate the results presented by Debien et al. [37], who showed an undulating characteristic in the training load dynamic over one season (36 weeks).

The adjustment between external intensity and workload distribution must be planned according to the phase of the competitive season [51]. Moreover, our findings provide coaches with information that will help them accurately implement periodization from a short-term perspective and adopt appropriate recovery strategies.

This study has some limitations. First, the data were obtained from a single volleyball team. In this way, specific trends and training periodization may be unique to this team. Second, training intensity [9] also considers the internal intensity [7] and its relationship [11, 56]; however, in this study, we intended to evaluate only the external training intensity. Third, we did not analyze or make comparisons with elite male athletes, which would have allowed us to present and discuss gender differences in training and match demands, thus contributing to the specific training planning. In addition, we did not consider contextual factors (e.g. situational variables) such as the importance of the match, athletes’ motivation, the moment of the season, or the team’s status in the championship. Considering such factors could elucidate the differences shown and the behavior of external intensity [53]. Finally, our study is the first to analyze this amount of external intensity variables, which made it difficult to discuss and compare the results of other studies. We suggest that future studies analyze these variables at different times of the season while observing players of different skill levels, age groups and sexes. In addition, studies are needed to verify the impact of the result and the importance of the match, daily recovery strategies and athletes’ well-being.

Our findings have practical applications and reinforce the need to make coaches and people involved with volleyball aware of the importance of controlling external training intensity. This daily control will help head coaches and coaching staff about a possible overload of the need to increase the training intensity. Volleyball training must result from the load demands while considering the specificity of the players and their specific tasks in the match [57]. Our results are very important, as we demonstrate that training must be monitored daily while considering each athlete’s playing position to balance training and match demands and avoid overload and possible injuries [3, 8, 50]. With this, the principle of individuality and training overload are respected and will benefit player performance. Another benefit of this study is that it provides methods for analyzing and quantifying external intensity. However, our results should be interpreted with caution, as they are dependent on the training program and the athletes who participated in this research.

## Conclusion

The present study highlighted the importance of monitoring training in volleyball, thus providing information that coaches can use to accurately implement periodization from a short-term perspective and appropriately adopt recovery strategies. Regarding the within-week differences in external training intensity in different microcycles, the intensity of all performance indicators – except for strain, acc/dec and acute mean load (RA) – showed significant differences depending on the distance to the match day, with moderate to large effect sizes. When scrutinizing by playing position, differences were found in load, with MB presenting lower loads when compared with other positions in the field.

## Electronic supplementary material

Below is the link to the electronic supplementary material.

Supplementary Material 1

## Data Availability

The data presented in this study are available on website: https://osf.io/6BRF4/ with Identifier: DOI 10.17605/OSF.IO/6BRF4.
