# Supplementary material for: Within-week differences in external training load demands in elite volleyball players
Source: BMC Sports Sci Med Rehabil. 2022 Nov 1;14:188. doi: 10.1186/s13102-022-00568-1 (PMC9628072; doi:10.1186/s13102-022-00568-1)
Supplement: Supplementary file 1 — Supplementary Material 1 [file 13102_2022_568_MOESM1_ESM.pdf]

SUPPLEMENTARY MATERIALS

Table S1. Mean, standard deviations and one way ANOVA results for training load metrics for MD+1 and MD-T across different positions.

Variables (Microcycle MD+1)	LH	L	MB	RH	S	F	p	Source of Difference*	Effect Size
Workload (A.U)	889.94 ± 306.45	870.50 ± 322.97	779.83 ± 297.74	885.38 ± 272.68	835.01 ± 328.56	2.21	0.068	-	0.022 (Small)
LPS Total Distance (m)	3884.17 ± 686.72	3107.00 ± 706.77	3139.45 ± 709.01	3635.62 ± 867.10	3376.78 ± 715.07	15.53	0.000	LH, RH - L, MB; S - LH	0.166 (Large)
LPS Jumps (N)	96.67 ± 30.17	9.84 ± 7.61	126.03 ± 47.36	118.98 ± 39.30	151.60 ± 63.40	81.40	0.000	MB, RH - L, LH, S; L - LH - S	0.504 (Large)
Acceleration (m/s^{-2})	145.38 ± 39.33	75.00 ± 26.70	101.24 ± 29.35	150.87 ± 37.79	97.36 ± 25.27	56.81	0.000	RH, LH - L, MB, S; L - MB, S	0.421 (Large)
Deceleration (m/s^{-2})	133.49 ± 37.47	81.04 ± 30.00	72.59 ± 23.83	134.60 ± 34.35	90.00 ± 25.25	63.23	0.000	RH, LH - L, MB, S; MB - S	0.448 (Large)
HMLD (m)	1255.57 ± 282.45	619.24 ± 188.86	928.16 ± 234.50	1272.6 ± 398.09	773.18 ± 223.13	63.95	0.000	RH, LH - L, MB, S; MB - L	0.450 (Large)
Acute Mean Load (EWMA)	963.75 ± 201.57	921.39 ± 191.40	851.84 ± 192.25	941.07 ± 185.22	885.81 ± 200.11	5.20	0.000	MB - LH, RH	0.051 (Small)
Chronic Mean Load (EWMA)	836.29 ± 131.90	803.52 ± 104.42	747.73 ± 122.75	817.07 ± 114.86	767.21 ± 133.28	8.39	0.000	LH - MB, S; RH - MB	0.080 (Moderate)
AC Ratio (EWMA)	1.16 ± 0.18	1.15 ± 0.19	1.14 ± 0.18	1.15 ± 0.17	1.16 ± 0.17	0.14	0.967	-	0.001 (Nonsig.)
Monotony (A.U)	1.43 ± 0.42	1.30 ± 0.38	1.44 ± 0.52	1.38 ± 0.34	1.37 ± 0.41	1.26	0.285	-	0.013 (Small)
Strain (A.U)	9337.82 ± 3785.35	8027.39 ± 3595.33	8240.77 ± 3867.72	8644.8 ± 3356.75	8291.25 ± 3477.12	1.84	0.120	-	0.019 (Small)
Stand_ Workload (A.U)	7.57 ± 1.38	7.17 ± 1.85	6.74 ± 1.39	7.47 ± 1.19	6.99 ± 1.53	5.29	0.000	MB - LH, RH	0.052 (Small)
Stand_LPS Total Distance (m/min)	34.02 ± 11.29	26.36 ± 7.69	28.79 ± 12.05	32.27 ± 11.66	29.30 ± 9.80	5.02	0.001	LH - L, MB	0.060 (Moderate)
Stand_LPS Jumps (N/min)	0.85 ± 0.36	0.08 ± 0.07	1.16 ± 0.58	1.06 ± 0.45	1.32 ± 0.62	52.28	0.000	S, MB - L, LH; RH - L, S; L - LH	0.394 (Large)
Stand_ Acceleration (N/min)	1.28 ± 0.50	0.64 ± 0.27	0.93 ± 0.41	1.34 ± 0.49	0.84 ± 0.31	27.45	0.000	RH, LH - L, MB, S; L - MB	0.260 (Large)

Stand_ Deceleration (N/min)	1.17 ± 0.46	0.69 ± 0.29	0.67 ± 0.31	1.19 ± 0.43	0.78 ± 0.30	32.30	0.000	LH, RH - L, MB, S	0.293 (Large)
Stand_ HMLD (m/min)	10.96 ± 3.81	5.27 ± 01.92	8.48 ± 03.53	11.25 ± 4.79	6.69 ± 02.56	29.89	0.000	RH, LH - L, MB, S; MB - L, S	0.277 (Large)
Accel Max (m/s^{-2})	2.94 ± 0.36	3.03 ± 0.25	2.69 ± 0.44	2.89 ± 0.46	3.15 ± 0.27	13.60	0.000	MB - LH, L, RH, S; S - LH, RH	0.148 (Large)
Decel Max (m/s^{-2})	-2.85 ± 0.39	-3.12 ± 0.36	-2.62 ± 0.50	-2.65 ± 0.39	-3.09 ± 0.43	16.72	0.000	RH, MB - L, LH, S; LH - L, S	0.176 (Large)
Max Speed(m/s^{-1})	17.52 ± 2.20	17.06 ± 1.92	15.42 ± 2.07	16.45 ± 2.27	17.42 ± 1.49	14.11	0.000	MB - LH, L, RH, S; RH - LH	0.153 (Large)
Acc/ Dec (m/s^{-2})	-1.04 ± 0.12	-0.98 ± 0.09	-1.05 ± 0.18	-1.09 ± 0.10	-1.04 ± 0.14	4.36	0.002	RH-L	0.053 (Small)
Accum Acce Load (A.U)	529.61 ± 126.11	484.08 ± 132.64	402.68 ± 121.59	500.38 ± 143.27	520.16 ± 160.62	11.69	0.000	MB - LH, L, RH, S; RH - L	0.130 (Moderate)
Acute Mean Load (RA)	907.89 ± 196.88	854.6 ± 185.19	794.23 ± 198.77	872.67 ± 186.79	844.05 ± 184.82	5.02	0.001	MB - LH	0.049 (Small)
Chronic Mean Load (RA)	811.05 ± 173.12	783.83 ± 138.44	731.85 ± 146.97	795.55 ± 149.81	743.97 ± 170.73	4.34	0.002	MB - LH	0.043 (Small)
AC Ratio (RA)	1.16 ± 0.32	1.11 ± 0.28	1.11 ± 0.31	1.12 ± 0.26	1.18 ± 0.33	0.844	0.498	-	0.009 (Nonsig.)

Variables (Microcycle MD-T)	LH	L	MB	RH	S	F	p	Source of Difference*	Effect Size
Workload (A.U)	294.34 ± 122.08	283.98 ± 129.94	331.79 ± 155.56	277.56 ± 116.71	256.46 ± 140.86	3.52	0.008	S - MB	0.035 (Small)
LPS Total Distance (m)	3010.90 ± 1106.39	2463.37 ± 1159.59	2536.69 ± 681.96	2946.95 ± 1348.81	2888 ± 983.51	1.57	0.187	-	0.047 (Small)
LPS Jumps (N)	66.27 ± 37.01	10.52 ± 5.54	73.01 ± 47.05	71.60 ± 51.69	93.08 ± 59.33	13.25	0.000	L - LH, MB, RH, S; RH - S	0.148 (Large)
Acceleration (m/s^{-2})	103.77 ± 53.34	59.21 ± 44.79	64.35 ± 34.84	103.15 ± 63.95	83.53 ± 33.68	5.29	0.001	LH, RH - L, MB	0.144 (Large)
Deceleration (m/s^{-2})	85.31 ± 50.50	60.26 ± 46.68	46.74 ± 24.71	87.10 ± 56.99	78.16 ± 32.52	4.83	0.001	MB - LH, RH	0.133 (Moderate)
HMLD (m)	1099.28 ± 363.83	641.11 ± 350.76	903.79 ± 243.00	1162.2 ± 572.18	815.42 ± 278.68	7.49	0.000	LH, RH - L, S	0.192 (Large)
Acute Mean Load (EWMA)	868.92 ± 214.16	807.01 ± 234.37	778.55 ± 220.86	848.64 ± 247.58	796.52 ± 190.77	2.85	0.024	MB - LH	0.029 (Small)

Chronic Mean Laod (EWMA)	848.42 ± 127.46	795.75 ± 129.12	737.65 ± 145.81	817.57 ± 146.84	780.15 ± 110.21	10.39	0.000	LH - MB, S; RH - MB	0.097 (Moderate)
AC Ratio (EWMA)	1.02 ± 0.18	1.00 ± 0.21	1.05 ± 0.21	1.03 ± 0.19	1.02 ± 0.20	0.70	0.594	-	0.007 (Nonsig.)
Monotony (A.U)	1.54 ± 0.35	1.45 ± 0.39	1.56 ± 0.45	1.56 ± 0.31	1.53 ± 0.35	0.82	0.513	-	0.008 (Nonsig.)
Strain (A.U)	9506.61 ± 3581.93	8265.96 ± 3792.12	8618.69 ± 3915.57	9323.07 ± 3539.47	8556.90 ± 2843.28	1.68	0.153	-	0.017 (Small)
Stand_Workload (A.U)	4.63 ± 1.18	4.48 ± 1.18	4.97 ± 1.24	4.38 ± 0.95	3.94 ± 1.15	7.86	0.000	MB - RH, S; LH - S	0.075 (Moderate)
Stand_LPS Total Distance (m/min)	48.17 ± 20.1	38.54 ± 19.1	40.99 ± 13.05	46.15 ± 22.4	45.78 ± 16.23	1.29	0.277	-	0.039 (Small)
Stand_LPS Jumps (N/min)	1.11 ± 0.67	0.17 ± 0.10	1.23 ± 0.83	1.20 ± 0.94	1.55 ± 1.02	11.76	0.000	L - LH, MB, RH, S; LH - S	0.134 (Moderate)
Stand_Acceleration (N/min)	1.67 ± 0.97	0.92 ± 0.76	1.04 ± 0.64	1.6 ± 1.06	1.32 ± 0.54	4.35	0.002	LH - L, MB	0.121 (Moderate)
Stand_Deceleration (N/min)	1.37 ± 0.91	0.93 ± 0.78	0.75 ± 0.45	1.36 ± 0.97	1.23 ± 0.51	4.02	0.004	MB - LH, RH	0.113 (Moderate)
Stand_HMLD (m/min)	17.92 ± 7.63	10.21 ± 6.29	14.97 ± 5.61	18.39 ± 10.07	13.18 ± 5.35	5.11	0.001	L - LH, RH	0.140 (Large)
Accel Max (m/s^{-2})	2.85 ± 0.42	2.85 ± 0.50	2.68 ± 0.51	2.73 ± 0.43	2.81 ± 0.31	0.84	0.504	-	0.026 (Small)
Decel Max (m/s^{-2})	-2.8 ± 0.57	-2.85 ± 0.58	-2.47 ± 0.45	-2.66 ± 0.53	-2.9 ± 0.52	3.01	0.021	S - MB	0.087 (Moderate)
Max Speed(m/s^{-1})	15.86 ± 2.08	15.44 ± 2.68	15.06 ± 1.92	15.17 ± 2.32	16.25 ± 1.67	1.35	0.257	-	0.041 (Small)
Acc/ Dec (m/s^{-2})	-1.05 ± 0.17	-1.01 ± 0.10	-1.10 ± 0.22	-1.05 ± 0.16	-0.99 ± 0.15	1.64	0.168	-	0.050 (Small)
Accum Acce Load (A.U)	413.26 ± 172.60	362.11 ± 166.17	319.23 ± 119.98	398.22 ± 197.82	401.59 ± 136.52	1.88	0.119	-	0.056 (Small)
Acute Mean Load (RA)	863.51 ± 196.98	796.18 ± 221.06	758.73 ± 206.03	837.94 ± 229.07	794.55 ± 180.80	4.12	0.003	MB - LH	0.041 (Small)
Chronic Mean Load (RA)	851.93 ± 158.71	802.59 ± 138.94	735.26 ± 162.32	813.08 ± 165.21	780.63 ± 148.52	8.28	0.000	MB - LH, RH; LH - S	0.079 (Moderate)
AC Ratio (RA)	1.04 ± 0.29	1.00 ± 0.29	1.05 ± 0.28	1.05 ± 0.30	1.05 ± 0.31	0.299	0.879	-	0.003 (Nonsig.)

LH: Left Hitter, RH: Right Hitter, MB: Middle Blocker, S: Setter and L: Libero

* Examples for Source of difference column: (A) – (B) denotes significant differences between A and B; (A)-(B)-(C) denotes significant differences for all possible pairwise combinations for A, B and C. (A)-(B),(C) denotes significant differences between A and B, and also between A and C, (A),(B) – (C) denotes significant differences between A and C, and also between B and C

Table S2. Mean, standard deviations and one way ANOVA results for training load metrics for MD and MD-1 across different positions.

Variables (Microcycle MD)	LH	L	MB	RH	S	F	p	Source of Difference*	Effect Size
Workload (A.U)	652.75 ± 466.46	578.30 ± 453.75	640.03 ± 446.59	596.28 ± 462.32	568.58 ± 404.51	1.14	0.335	-	0.006 (Nonsig.)
LPS Total Distance (m)	3003.12 ± 1051.52	2357.51 ± 1010.82	2414.31 ± 688.06	2929.57 ± 1127.31	2788.6 ± 991.84	7.91	0.000	LH, RH - L, MB	0.075 (Moderate)
LPS Jumps (N)	67.09 ± 33.49	8.52 ± 5.84	76.26 ± 44.35	72.01 ± 44.65	98.82 ± 58.85	47.65	0.000	L, S - LH, MB, RH; S - L	0.211 (Large)
Acceleration (m/s^{-2})	103.18 ± 48.73	52.53 ± 36.68	63.39 ± 31.87	102.4 ± 51.26	82.66 ± 35.52	23.46	0.000	MB, L - LH, RH, S; LH - S	0.194 (Large)
Deceleration (m/s^{-2})	86.31 ± 46.22	51.86 ± 37.34	45.10 ± 22.98	85.42 ± 46.50	76.38 ± 32.66	22.44	0.000	LH, RH, S - L, MB	0.187 (Large)
HMLD (m)	1061.21 ± 358.53	574.34 ± 303.42	831.57 ± 264.17	1136.03 ± 450.86	758.88 ± 280.54	31.74	0.000	RH, LH - L, MB, S; L - MB, S	0.246 (Large)
Acute Mean Load (EWMA)	860.94 ± 210.04	801.36 ± 210.38	786.11 ± 202.8	821.08 ± 222.86	783.70 ± 191.95	4.74	0.001	LH - MB, S	0.023 (Small)
Chronic Mean Load (EWMA)	835.34 ± 120.70	787.32 ± 126.87	745.81 ± 121.83	802.52 ± 141.61	765.77 ± 122.97	15.67	0.000	LH - L, MB, S; MB - L, RH	0.072 (Moderate)
AC Ratio (EWMA)	1.03 ± 0.18	1.01 ± 0.18	1.05 ± 0.19	1.02 ± 0.17	1.02 ± 0.18	1.18	0.320	-	0.006 (Nonsig.)
Monotony (A.U)	1.48 ± 0.43	1.38 ± 0.39	1.54 ± 0.63	1.46 ± 0.33	1.42 ± 0.34	2.63	0.033	MB - L	0.013 (Small)
Strain (A.U)	9150.71 ± 4073.79	7981.31 ± 3685.44	8598.34 ± 4438.26	8625.36 ± 3502.62	7967.28 ± 3044.02	2.71	0.029	S - LH	0.013 (Small)
Stand_Workload (A.U)	5.67 ± 2.76	5.07 ± 2.68	5.41 ± 2.53	5.17 ± 2.59	4.96 ± 2.42	2.05	0.086	-	0.010 (Nonsig.)
Stand_LPS Total Distance (m/min)	30.88 ± 10.84	24.16 ± 8.30	25.13 ± 9.66	30.55 ± 9.21	28.29 ± 10.17	7.92	0.000	LH, RH - L, MB	0.075 (Moderate)
Stand_LPS Jumps (N/min)	0.73 ± 0.33	0.12 ± 0.13	0.73 ± 0.27	0.73 ± 0.27	0.97 ± 0.44	75.70	0.000	L, S - LH, MB, RH; L - S	0.299 (Large)
Stand_Acceleration (N/min)	1.00 ± 0.36	0.49 ± 0.26	0.59 ± 0.19	1.00 ± 0.31	0.81 ± 0.31	51.43	0.000	RH, LH - L, MB, S; S - L, MB	0.346 (Large)

Stand_Deceleration (N/min)	0.80 ± 0.31	0.47 ± 0.26	0.43 ± 0.16	0.82 ± 0.28	0.75 ± 0.28	43.62	0.000	L, MB - LH, RH, S	0.310 (Large)
Stand_HMLD (m/min)	11.29 ± 5.06	5.79 ± 2.44	9.00 ± 4.68	11.94 ± 4.13	8.03 ± 4.34	21.65	0.000	L - MB, S; RH, LH - L, MB, S	0.182 (Large)
Accel Max (m/s^{-2})	2.77 ± 0.38	2.88 ± 0.43	2.59 ± 0.49	2.73 ± 0.38	2.80 ± 0.34	5.38	0.000	MB - LH, L, S	0.052 (Small)
Decel Max (m/s^{-2})	-2.76 ± 0.56	-2.92 ± 0.52	-2.42 ± 0.51	-2.61 ± 0.44	-2.82 ± 0.44	11.93	0.000	MB - LH, L, S; L - RH	0.109 (Moderate)
Max Speed(m/s^{-1})	15.53 ± 2.29	15.59 ± 2.32	14.65 ± 1.95	15.17 ± 1.98	15.89 ± 1.76	4.35	0.002	MB - LH, S	0.043 (Small)
Acc/ Dec (m/s^{-2})	-1.02 ± 0.15	-1.00 ± 0.12	-1.10 ± 0.23	-1.06 ± 0.15	-1.01 ± 0.13	4.71	0.001	MB - LH, L, S	0.046 (Small)
Accum Acce Load (A.U)	411.04 ± 152.45	348.92 ± 135.56	310.60 ± 103.17	399.25 ± 153.73	397.41 ± 139.74	9.06	0.000	L - LH, MB - LH, RH, S	0.085 (Moderate)
Acute Mean Load (RA)	861.36 ± 193.90	804.07 ± 196.11	775.24 ± 185.87	822.18 ± 208.92	785.17 ± 185.12	6.46	0.000	LH - MB, S	0.031 (Small)
Chronic Mean Load (RA)	835.93 ± 152.12	787.63 ± 146.14	740.02 ± 143.63	801.73 ± 165.55	767.47 ± 158.53	11.92	0.000	MB - LH, L, RH; LH - L, S	0.056 (Small)
AC Ratio (RA)	1.06 ± 0.29	1.04 ± 0.28	1.07 ± 0.29	1.05 ± 0.27	1.06 ± 0.30	0.303	0.876	-	0.002 (Nonsig.)

Variables (Microcycle MD-1)	LH	L	MB	RH	S	F	p	Source of Difference*	Effect Size
Workload (A.U)	742.72 ± 227.63	696.13 ± 267.70	677.50 ± 219.75	723.31 ± 233.54	712.67 ± 243.23	2.82	0.024	MB - LH	0.012 (Small)
LPS Total Distance (m)	3384.78 ± 692.42	2832.41 ± 649.57	2889.5 ± 720.75	3392.79 ± 796.82	3109.77 ± 774.18	18.53	0.000	LH, RH - L, MB; LH - S	0.100 (Moderate)
LPS Jumps (N)	80.44 ± 23.46	9.87 ± 9.72	110.39 ± 34.10	98.42 ± 29.05	126.47 ± 48.06	228.40	0.000	LH - L - MB - RH - S	0.532 (Large)
Acceleration (m/s^{-2})	120.36 ± 38.02	69.87 ± 21.71	94.28 ± 32.09	129.24 ± 38.29	96.4 ± 28.45	56.27	0.000	L - MB, S; RH, LH - L, MB, S	0.253 (Large)
Deceleration (m/s^{-2})	108.48 ± 36.38	70.24 ± 22.83	67.83 ± 24.95	115.93 ± 36.02	84.79 ± 29.07	70.55	0.000	RH, LH - L, MB, S; S - L, MB	0.298 (Large)
HMLD (m)	1114.13 ± 297.48	579.82 ± 227.33	886.7 ± 269.12	1213.8 ± 382.93	735.87 ± 246.74	90.00	0.000	LH - L - MB - RH - S	0.351 (Large)
Acute Mean Load (EWMA)	918.07 ± 197.69	871.17 ± 198.67	814.59 ± 184.71	883.88 ± 192.71	854.74 ± 193.78	10.05	0.000	MB - LH, L, RH; S - LH	0.041 (Small)
Chronic Mean Load (EWMA)	832.55 ± 127.03	795.81 ± 120.52	740.01 ± 118.82	806.68 ± 131.85	766.77 ± 131.30	20.19	0.000	L - LH, MB, S; LH - MB, S; RH - MB	0.080 (Moderate)

AC Ratio (EWMA)	1.11 ± 0.18	1.09 ± 0.19	1.10 ± 0.18	1.10 ± 0.16	1.12 ± 0.19	0.46	0.765	-	0.002 (Nonsig.)
Monotony (A.U)	1.42 ± 0.40	1.36 ± 0.38	1.49 ± 0.57	1.43 ± 0.37	1.39 ± 0.38	2.08	0.082	-	0.009 (Nonsig.)
Strain (A.U)	8889.28 ± 3602.89	8189.21 ± 3551.19	8322.85 ± 3925.60	8617.18 ± 3363.67	8061.65 ± 3354.66	1.70	0.148	-	0.007 (Nonsig.)
Stand_Workload (A.U)	7.16 ± 1.33	6.49 ± 1.74	6.53 ± 1.27	6.93 ± 1.25	6.73 ± 1.26	9.47	0.000	LH - L, MB, S; RH - MB	0.039 (Small)
Stand_LPS Total Distance (m/min)	32.5 ± 8.02	26.54 ± 7.54	28.62 ± 9.21	32.69 ± 8.55	30.07 ± 9.4	11.44	0.000	LH, RH - L, MB; L - S	0.064 (Moderate)
Stand_LPS Jumps (N/min)	0.79 ± 0.24	0.09 ± 0.09	1.10 ± 0.39	0.97 ± 0.30	1.22 ± 0.48	201.10	0.000	LH - L - MB - RH - S	0.500 (Large)
Stand_Acceleration (N/min)	1.15 ± 0.38	0.65 ± 0.21	0.93 ± 0.37	1.25 ± 0.41	0.93 ± 0.31	46.06	0.000	L - MB, S; RH, LH - L, MB, S	0.217 (Large)
Stand_Deceleration (N/min)	1.04 ± 0.37	0.66 ± 0.22	0.67 ± 0.28	1.12 ± 0.39	0.82 ± 0.31	56.36	0.000	RH, LH - L, MB, S; S - L, MB	0.253 (Large)
Stand_HMLD (m/min)	10.71 ± 3.15	5.4 ± 2.09	8.76 ± 3.10	11.72 ± 4.00	7.09 ± 2.55	75.99	0.000	L - MB, S; RH, LH - L, MB, S; MB - S	0.314 (Large)
Accel Max (m/s^{-2})	2.88 ± 0.36	3.13 ± 0.32	2.73 ± 0.41	2.87 ± 0.38	3.01 ± 0.47	19.81	0.000	L - LH, RH; MB - LH, L, RH, S	0.106 (Moderate)
Decel Max (m/s^{-2})	-2.81 ± 0.40	-3.13 ± 0.37	-2.63 ± 0.50	-2.67 ± 0.45	-2.93 ± 0.54	23.46	0.000	L - LH, MB, RH, S; MB - LH; S - MB, RH	0.123 (Moderate)
Max Speed(m/s^{-1})	16.53 ± 2.17	17.12 ± 1.99	15.83 ± 2.06	16.24 ± 2.25	16.69 ± 2.07	7.05	0.000	MB - LH, L, S; RH - L	0.041 (Small)
Acc/ Dec (m/s^{-2})	-1.04 ± 0.11	-1.01 ± 0.10	-1.06 ± 0.17	-1.09 ± 0.12	-1.05 ± 0.15	4.82	0.001	L - MB, RH; LH - RH	0.028 (Small)
Accum Acce Load (A.U)	447.18 ± 119.54	416.83 ± 105.37	347.53 ± 102.42	439.31 ± 130.01	439.87 ± 146.00	20.87	0.000	MB - LH, L, RH, S	0.111 (Moderate)
Acute Mean Load (RA)	875.01 ± 186.40	836.2 ± 191.59	782.29 ± 177.36	844.58 ± 180.97	809.24 ± 185.36	9.23	0.000	MB - LH, L, RH; S - LH	0.038 (Small)
Chronic Mean Load (RA)	813.51 ± 166.16	777.25 ± 145.86	721.84 ± 145.05	788.03 ± 163.62	748.77 ± 169.71	12.46	0.000	MB - LH, L, RH; S - LH	0.051 (Small)
AC Ratio (RA)	1.12 ± 0.32	1.10 ± 0.32	1.12 ± 0.32	1.10 ± ´0.28	1.13 ± 0.34	0.14	0.967	-	0.001 (Nonsig.)

LH: Left Hitter, RH: Right Hitter, MB: Middle Blocker, S: Setter and L: Libero

* Examples for Source of difference column: (A) – (B) denotes significant differences between A and B; (A)-(B)-(C) denotes significant differences for all possible pairwise combinations for A, B and C. (A)-(B),(C) denotes significant differences between A and B, and also between A and C, (A),(B) – (C) denotes significant differences between A and C, and also between B and C

Table S3. Mean, standard deviations and one way ANOVA results for training load metrics for MD-2 and MD-3 across different positions.

Variables (Microcycle MD-2)	LH	L	MB	RH	S	F	p	Source of Difference*	Effect Size
Workload (A.U)	942.77 ± 288.13	891.66 ± 297.26	828.60 ± 289.02	898.90 ± 318.52	903.98 ± 295.33	2.81	0.025	MB - LH	0.021 (Small)
LPS Total Distance (m)	3960.80 ± 792.63	3384.15 ± 663.000	3265.08 ± 627.62	4063.52 ± 752.88	3548.13 ± 753.12	24.58	0.000	LH, RH - L, MB, S	0.171 (Large)
LPS Jumps (N)	95.34 ± 25.03	12.02 ± 11.24	132.44 ± 31.60	117.55 ± 24.82	137.87 ± 48.44	218.60	0.000	LH - L - RH; MB, S - LH, L, RH	0.641 (Large)
Acceleration (m/s^{-2})	144.70 ± 41.90	84.07 ± 25.52	107.63 ± 31.57	162.47 ± 34.13	106.38 ± 31.47	69.19	0.000	LH - L - RH; MB, S - LH, L, RH	0.367 (Large)
Deceleration (m/s^{-2})	129.97 ± 37.29	87.94 ± 23.60	77.97 ± 23.87	146.86 ± 35.25	96.23 ± 30.01	86.98	0.000	LH, RH - L, MB, S; LH - RH; MB - S	0.421 (Large)
HMLD (m)	1300.61 ± 329.78	701.65 ± 269.52	971.44 ± 279.77	1414.52 ± 354.9	812.03 ± 293.93	82.14	0.000	RH, LH - L, MB, S; MB - L, S	0.407 (Large)
Acute Mean Load (EWMA)	973.80 ± 178.82	926.61 ± 181.18	848.91 ± 156.30	925.85 ± 200.59	898.84 ± 199.34	9.18	0.000	MB - LH, L, RH; S - LH	0.067 (Moderate)
Chronic Mean Load (EWMA)	843.58 ± 122.81	802.73 ± 128.78	750.44 ± 105.54	808.11 ± 143.80	770.39 ± 136.88	11.29	0.000	MB - LH, L, RH; S - LH	0.081 (Moderate)
AC Ratio (EWMA)	1.16 ± 0.16	1.15 ± 0.17	1.13 ± 0.14	1.15 ± 0.16	1.17 ± 0.18	0.83	0.506	-	0.006 (Nonsig.)
Monotony (A.U)	1.33 ± 0.28	1.29 ± 0.29	1.39 ± 0.30	1.33 ± 0.28	1.28 ± 0.29	2.55	0.039	MB - S	0.020 (Small)
Strain (A.U)	8600.82 ± 3200.16	8015.76 ± 3161.66	7874.94 ± 2983.41	8218 ± 3254.33	7668.07 ± 3343.5	1.50	0.202	-	0.012 (Small)
Stand_Workload (A.U)	7.79 ± 1.36	7.23 ± 1.49	6.97 ± 1.43	7.53 ± 1.42	7.38 ± 1.43	6.61	0.000	LH - L, MB; RH - MB	0.049 (Small)
Stand_LPS Total Distance (m/min)	33.52 ± 7.58	29.09 ± 13.78	28.87 ± 8.48	46.84 ± 101.54	29.6 ± 7.09	2.81	0.025	RH - MB	0.023 (Small)
Stand_LPS Jumps (N/min)	0.81 ± 0.23	0.11 ± 0.10	1.18 ± 0.39	1.35 ± 2.82	1.16 ± 0.42	14.58	0.000	LH, L - MB, RH; L - LH; S - L	0.106 (Moderate)
Stand_Acceleration (N/min)	1.22 ± 0.39	0.73 ± 0.42	0.95 ± 0.36	1.87 ± 3.96	0.88 ± 0.26	6.12	0.000	RH - LH, L, MB, S	0.049 (Small)
Stand_Deceleration (N/min)	1.09 ± 0.34	0.76 ± 0.41	0.69 ± 0.26	1.65 ± 3.34	0.80 ± 0.26	7.43	0.000	MB - LH, RH - LH, L, MB, S	0.059 (Moderate)
Stand_HMLD (m/min)	11.06 ± 3.24	6.03 ± 3.45	8.57 ± 3.07	16.42 ± 35.89	6.76 ± 2.46	6.53	0.000	RH - L, MB, S	0.052 (Small)

Accel Max (m/s^{-2})	2.98 ± 0.35	3.20 ± 0.29	2.77 ± 0.45	2.90 ± 0.37	2.97 ± 0.35	15.55	0.000	L - LH, MB, RH, S; MB - LH, S	0.115 (Moderate)
Decel Max (m/s^{-2})	-2.82 ± 0.40	-3.22 ± 0.39	-2.71 ± 0.51	-2.68 ± 0.35	-2.93 ± 0.49	18.74	0.000	L - LH, MB, RH; S - L, MB, RH	0.136 (Moderate)
Max Speed(m/s^{-1})	17.09 ± 2.03	17.63 ± 1.87	16.35 ± 2.16	16.96 ± 2.29	17.18 ± 1.99	4.91	0.001	MB - LH, L	0.039 (Small)
Acc/ Dec (m/s^{-2})	-1.07 ± 0.14	-1.00 ± 0.09	-1.04 ± 0.17	-1.09 ± 0.11	-1.03 ± 0.15	4.69	0.001	L - LH, RH	0.038 (Small)
Accum Acce Load (A.U)	512.60 ± 116.58	480.71 ± 100.89	388.72 ± 82.16	525.44 ± 101.86	499.76 ± 122.11	32.34	0.000	MB - LH, L, RH, S	0.213 (Large)
Acute Mean Load (RA)	889.88 ± 194.57	855.05 ± 205.72	784.42 ± 163.61	850.90 ± 206.72	818.98 ± 206.54	5.96	0.000	MB - LH	0.044 (Small)
Chronic Mean Load (RA)	830.77 ± 141.48	784.32 ± 152.15	735.78 ± 120.96	793.77 ± 155.54	758.67 ± 149.82	8.99	0.000	LH - MB, S; RH - MB	0.066 (Moderate)
AC Ratio (RA)	1.08 ± 0.26	1.12 ± 0.33	1.09 ± 0.28	1.08 ± 0.24	1.09 ± 0.27	0.233	0.92	-	0.002 (Nonsig.)

Variables (Microcycle MD-3)	LH	L	MB	RH	S	F	p	Source of Difference*	Effect Size
Workload (A.U)	910.00 ± 336.15	873.37 ± 324.03	756.95 ± 301.37	861.19 ± 349.48	849.63 ± 329.73	3.69	0.006	LH - MB	0.033 (Small)
LPS Total Distance (m)	4112.76 ± 786.21	3544.36 ± 613.48	3481.98 ± 820.24	3976.41 ± 985.95	3621.50 ± 692.15	11.44	0.000	LH, RH - L, MB, S	0.106 (Moderate)
LPS Jumps (N)	100.60 ± 20.77	11.42 ± 8.25	134.02 ± 33.15	121.10 ± 34.26	161.50 ± 60.95	183.80	0.000	LH - L - S; RH, MB - L, LH, S	0.635 (Large)
Acceleration (m/s-2)	152.13 ± 37.10	88.25 ± 22.77	108.77 ± 30.67	157.38 ± 51.41	105.56 ± 28.54	54.11	0.000	LH, RH - L, MB, S; L - MB	0.360 (Large)
Deceleration (m/s-2)	144.02 ± 36.41	92.64 ± 23.24	79.83 ± 23.74	142.05 ± 48.09	94.89 ± 28.08	74.90	0.000	LH, RH - L, MB, S; MB - S	0.438 (Large)
HMLD (m)	1310.80 ± 339.90	718.73 ± 229.9	1019.24 ± 355.45	1314.96 ± 437.54	796.33 ± 198.51	47.92	0.000	LH, RH - L, MB, S; MB - L, S	0.332 (Large)
Acute Mean Load (EWMA)	1006.02 ± 171.63	966.31 ± 166.33	874.81 ± 152.1	949.36 ± 220.84	950.62 ± 198.87	8.81	0.000	MB - LH, L	0.076 (Moderate)
Chronic Mean Load (EWMA)	839.31 ± 136.10	800.33 ± 146.53	740.71 ± 120.98	801.39 ± 174.65	790.26 ± 146.03	7.75	0.000	MB - LH, RH	0.067 (Moderate)
AC Ratio (EWMA)	1.20 ± 0.16	1.21 ± 0.16	1.19 ± 0.14	1.19 ± 0.18	1.20 ± 0.20	0.40	0.806	-	0.004 (Nonsig.)

Monotony (A.U)	1.33 ± 0.34	1.31 ± 0.31	1.43 ± 0.32	1.35 ± 0.37	1.34 ± 0.38	1.87	0.116	-	0.017 (Small)
Strain (A.U)	9001.76 ± 3892.07	8492.82 ± 3394.94	8517.35 ± 3356.27	8837.14 ± 4150.19	8627.18 ± 3926.91	0.36	0.840	-	0.003 (Nonsig.)
Stand_Workload (A.U)	7.70 ± 1.40	7.21 ± 1.49	6.58 ± 1.23	7.37 ± 1.40	7.16 ± 1.30	11.06	0.000	MB - LH, L, RH	0.093 (Moderate)
Stand_LPS Total Distance (m/min)	37.61 ± 14.36	31.03 ± 10.39	33.41 ± 14.19	36.67 ± 14.18	33.35 ± 12.78	3.00	0.019	L - LH	0.030 (Small)
Stand_LPS Jumps (N/min)	0.95 ± 0.39	0.11 ± 0.10	1.33 ± 0.64	1.17 ± 0.54	1.51 ± 0.70	71.64	0.000	LH - MB, S; L - LH, MB, RH, S; S - RH	0.404 (Large)
Stand_Acceleration (N/min)	1.39 ± 0.58	0.77 ± 0.30	1.04 ± 0.47	1.44 ± 0.67	0.96 ± 0.39	21.76	0.000	RH, LH - L, MB, S; L - MB	0.184 (Large)
Stand_Deceleration (N/min)	1.32 ± 0.57	0.81 ± 0.32	0.76 ± 0.35	1.29 ± 0.59	0.87 ± 0.37	29.60	0.000	LH, RH - L, MB, S	0.235 (Large)
Stand_HMLD (m/min)	11.96 ± 4.87	6.27 ± 2.49	9.70 ± 4.43	12.05 ± 5.40	7.34 ± 2.95	24.44	0.000	RH, LH - L, MB, S; MB - L, S	0.203 (Large)
Accel Max (m/s-2)	2.98 ± 0.37	3.21 ± 0.48	2.8 ± 0.41	2.72 ± 0.40	2.97 ± 0.31	14.43	0.000	LH - MB, RH; L - LH, MB, RH, S; RH -S	0.130 (Moderate)
Decel Max (m/s-2)	-2.96 ± 0.38	-3.27 ± 0.44	-2.74 ± 0.52	-2.62 ± 0.34	-2.91 ± 0.48	20.07	0.000	LH - MB, RH; L - LH, MB, RH, S; RH -S	0.173 (Large)
Max Speed(m/s-1)	17.57 ± 2.22	17.96 ± 2.27	16.71 ± 1.84	16.19 ± 2.89	16.95 ± 2.03	6.67	0.000	LH, L - MB, RH	0.065 (Moderate)
Acc/ Dec (m/s-2)	-1.02 ± 0.14	-1.00 ± 0.20	-1.04 ± 0.13	-1.05 ± 0.13	-1.03 ± 0.12	1.30	0.269	-	0.013 (Small)
Accum Acce Load (A.U)	537.69 ± 108.70	501.21 ± 82.65	415.9 ± 89.47	522.61 ± 123.75	565.67 ± 279.92	15.42	0.000	MB - LH, L, RH, S	0.138 (Large)
Acute Mean Load (RA)	919.25 ± 222.86	888.77 ± 212.21	822.71 ± 176.49	873.35 ± 256.1	865.71 ± 234.15	3.23	0.013	MB - LH	0.029 (Small)
Chronic Mean Load (RA)	819.23 ± 157.32	778.61 ± 161.80	724.67 ± 129.61	782.53 ± 185.57	770.73 ± 166.24	5.79	0.000	MB - LH	0.051 (Small)
AC Ratio (RA)	1.14 ± 0.29	1.17 ± 0.34	1.15 ± 0.23	1.12 ± 0.29	1.13 ± 0.34	0.235	0.918	-	0.002 (Nonsig.)

LH: Left Hitter, RH: Right Hitter, MB: Middle Blocker, S: Setter and L: Libero

* Examples for Source of difference column: (A) – (B) denotes significant differences between A and B; (A)-(B)-(C) denotes significant differences for all possible pairwise combinations for A, B and C. (A)-(B),(C) denotes significant differences between A and B, and also between A and C, (A),(B) – (C) denotes significant differences between A and C, and also between B and C

Table S4. Mean, standard deviations and one way ANOVA results for training load metrics for MD-4 and MD-5 across different positions.

Variables (Microcycle MD-4)	LH	L	MB	RH	S	F	p	Source of Difference*	Effect Size
Workload (A.U)	1038.8 ± 226.85	949.23 ± 246.29	884.36 ± 297.76	967.58 ± 241.01	1045.03 ± 250.46	3.63	0.007	MB - LH, S	0.062 (Moderate)
LPS Total Distance (m)	4783.85 ± 1651.86	3924.48 ± 1461.04	4126.00 ± 1591.70	4591.00 ± 1544.38	4319.28 ± 1392.07	1.91	0.111	-	0.042 (Small)
LPS Jumps (N)	93.87 ± 21.94	13.74 ± 9.03	121.16 ± 52.45	112.48 ± 38.65	143.44 ± 57.26	44.06	0.000	LH - MB, S; L - LH, MB, RH, S; RH - S	0.499 (Large)
Acceleration (m/s^{-2})	144.25 ± 29.74	77.33 ± 14.91	104.26 ± 28.51	153.23 ± 42.66	112.84 ± 25.37	35.13	0.000	LH, RH - L, MB, S; L - MB, S	0.448 (Large)
Deceleration (m/s^{-2})	131.53 ± 28.87	80.33 ± 19.36	77.49 ± 24.58	130.96 ± 37.04	99.32 ± 18.16	37.81	0.000	LH, RH - L, MB, S; RH - S	0.466 (Large)
HMLD (m)	1455.94 ± 279.69	730.56 ± 218.07	1077.30 ± 275.86	1360.65 ± 483.01	931.80 ± 234.69	33.31	0.000	LH, RH - L, MB, S; L - MB	0.435 (Large)
Acute Mean Load (EWMA)	856.51 ± 208.39	812.35 ± 186.13	741.03 ± 173.4	804.07 ± 219.49	839.39 ± 197.06	3.00	0.019	LH - MB	0.052 (Small)
Chronic Mean Load (EWMA)	801.71 ± 129.19	766.64 ± 129.47	701.61 ± 128.60	766.79 ± 167.73	770.68 ± 127.36	4.53	0.002	LH - MB	0.076 (Moderate)
AC Ratio (EWMA)	1.06 ± 0.20	1.06 ± 0.16	1.06 ± 0.15	1.04 ± 0.15	1.09 ± 0.18	0.34	0.852	-	0.006 (Nonsig.)
Monotony (A.U)	1.28 ± 0.35	1.28 ± 0.33	1.37 ± 0.32	1.33 ± 0.36	1.33 ± 0.33	0.74	0.568	-	0.013 (Small)
Strain (A.U)	8309.94 ± 4081.41	7707.56 ± 3577.59	7762.68 ± 3554.24	8210.02 ± 4242.29	8343.07 ± 3943.60	0.28	0.890	-	0.005 (Nonsig.)
Stand_Workload (A.U)	7.73 ± 1.14	6.97 ± 1.44	6.84 ± 1.41	7.42 ± 0.94	7.61 ± 1.09	5.38	0.000	LH - L, MB; MB -S	0.089 (Moderate)
Stand_LPS Total Distance (m/min)	36.44 ± 12.36	30.12 ± 11.13	34.26 ± 14.5	37.57 ± 14.78	33.11 ± 10.99	1.50	0.205	-	0.033 (Small)
Stand_LPS Jumps (N/min)	0.72 ± 0.15	0.11 ± 0.08	0.98 ± 0.36	0.91 ± 0.30	1.10 ± 0.44	52.10	0.000	LH -MB, S; L - LH, MB, RH, S	0.541 (Large)
Stand_Acceleration (N/min)	1.10 ± 0.25	0.60 ± 0.17	0.85 ± 0.24	1.25 ± 0.43	0.87 ± 0.26	25.33	0.000	LH, RH - L, MB, S; L - MB, S	0.369 (Large)
Stand_Deceleration (N/min)	1.00 ± 0.23	0.62 ± 0.19	0.64 ± 0.21	1.07 ± 0.38	0.76 ± 0.18	26.24	0.000	LH, RH - L, MB, S	0.378 (Large)
Stand_HMLD (m/min)	11.16 ± 2.52	5.68 ± 2.02	8.94 ± 2.77	11.08 ± 4.54	7.19 ± 2.17	22.39	0.000	LH, RH - L, MB, S; L - MB	0.341 (Large)

Accel Max (m/s^{-2})	3.09 ± 0.36	3.08 ± 0.31	2.81 ± 0.44	2.94 ± 0.47	3.19 ± 0.37	5.25	0.001	MB - LH, L, S	0.108 (Moderate)
Decel Max (m/s^{-2})	-3.00 ± 0.46	-3.26 ± 0.29	-2.64 ± 0.43	-2.66 ± 0.42	-3.13 ± 0.46	13.68	0.000	L - LH; MB, RH - LH, L, S	0.240 (Large)
Max Speed(m/s^{-1})	18.44 ± 2.54	18.20 ± 2.14	16.93 ± 2.01	17.62 ± 3.45	18.11 ± 2.32	2.60	0.038	LH - MB	0.057 (Small)
Acc/ Dec (m/s^{-2})	-1.05 ± 0.14	-0.95 ± 0.08	-1.07 ± 0.14	-1.12 ± 0.16	-1.03 ± 0.13	5.88	0.000	L - LH, MB, RH	0.120 (Moderate)
Accum Acce Load (A.U)	534.06 ± 111.07	493.27 ± 95.27	418.19 ± 117.76	541.8 ± 113.42	535.72 ± 130.45	8.75	0.000	MB - LH, RH, S	0.168 (Large)
Acute Mean Load (RA)	867.07 ± 266.91	818.63 ± 236.54	768.15 ± 215.03	829.07 ± 277.09	849.4 ± 242.14	1.37	0.245	-	0.024 (Small)
Chronic Mean Load (RA)	819.24 ± 146.06	788.94 ± 139.19	723.85 ± 138.72	789.42 ± 173.52	793.14 ± 149.75	3.50	0.009	LH - MB	0.060 (Moderate)
AC Ratio (RA)	1.06 ± 0.32	1.04 ± 0.26	1.06 ± 0.24	1.04 ± 0.28	1.08 ± 0.28	0.117	0.976	-	0.002 (Nonsig.)

Variables (Microcycle MD-5)	LH	L	MB	RH	S	F	P-Value	Source of Difference*	Effect Size
Workload (A.U)	953.90 ± 298.74	1037.50 ± 289.87	707.23 ± 258.46	777.33 ± 373.65	957.93 ± 291.61	5.00	0.001	MB - LH, L	0.164 (Large)
LPS Total Distance (m)	3884.38 ± 608.89	3263.25 ± 536.21	3239.70 ± 314.32	4192.56 ± 394.11	3795.00 ± 251.72	12.22	0.000	L - LH, RH; MB - LH, RH, S	0.411 (Large)
LPS Jumps (N)	90.84 ± 24.68	6.50 ± 3.89	110.47 ± 43.89	128.60 ± 35.49	159.17 ± 66.97	18.86	0.000	L - LH, MB, RH, S; S - LH, MB	0.479 (Large)
Acceleration (m/s-2)	136.62 ± 35.66	80.88 ± 9.34	108.15 ± 35.93	166.89 ± 35.65	100.7 ± 26.67	10.41	0.000	LH, RH - L, MB, S	0.373 (Large)
Deceleration (m/s-2)	124.33 ± 42.95	80.25 ± 12.49	75.78 ± 28.57	151.00 ± 41.13	82.7 ± 18.04	13.36	0.000	LH, RH - L, MB, S	0.433 (Large)
HMLD (m)	1229.71 ± 154.13	646 ± 127.12	964.82 ± 206.01	1469.11 ± 267.45	840.60 ± 86.96	31.51	0.000	LH, RH - L, MB, S; LH - RH; L - MB	0.643 (Large)
Acute Mean Load (EWMA)	861.06 ± 236.04	827.37 ± 240.88	736.23 ± 215.80	783.56 ± 371.60	896.42 ± 207.89	1.54	0.197	-	0.057 (Small)
Chronic Mean Laod (EWMA)	776.63 ± 170.02	708.47 ± 196.12	666.40 ± 156.50	728.62 ± 244.93	779.39 ± 145.85	1.90	0.116	-	0.069 (Moderate)
AC Ratio (EWMA)	1.10 ± 0.15	1.17 ± 0.17	1.09 ± 0.13	1.00 ± 0.29	1.14 ± 0.12	2.21	0.073	-	0.080 (Moderate)

Monotony (A.U)	1.37 ± 0.63	1.38 ± 0.72	1.49 ± 0.74	1.42 ± 0.64	1.66 ± 0.70	0.48	0.754	-	0.018 (Small)
Strain (A.U)	9479.77 ± 6155.01	8434.01 ± 6165.94	8832.88 ± 6459.14	9264.99 ± 6636.67	11604.46 ± 6238.00	0.58	0.680	-	0.022 (Small)
Stand_Workload (A.U)	7.66 ± 1.42	8.21 ± 1.25	6.54 ± 1.48	7.10 ± 1.69	7.71 ± 1.05	4.76	0.001	MB - LH, L	0.157 (Large)
Stand_LPS Total Distance (m/min)	35.28 ± 11.59	26.05 ± 3.50	33.62 ± 12.22	41.33 ± 14.70	36.47 ± 15.12	1.78	0.142	-	0.093 (Moderate)
Stand_LPS Jumps (N/min)	0.8 ± 0.33	0.05 ± 0.03	1.10 ± 0.47	1.22 ± 0.49	1.53 ± 1.10	10.15	0.000	L - LH, MB, RH, S; LH - S	0.331 (Large)
Stand_Acceleration (N/min)	1.21 ± 0.35	0.65 ± 0.08	1.10 ± 0.49	1.61 ± 0.54	0.93 ± 0.32	6.45	0.000	LH - L; RH - L, MB, S	0.269 (Large)
Stand_Deceleration (N/min)	1.10 ± 0.41	0.64 ± 0.08	0.76 ± 0.33	1.43 ± 0.43	0.77 ± 0.24	9.67	0.000	LH - L, MB; RH - L, MB, S	0.356 (Large)
Stand_HMLD (m/min)	11.28 ± 3.56	5.42 ± 2.19	10.20 ± 4.65	14.96 ± 7.20	8.18 ± 3.77	5.67	0.001	LH - L, RH - L, S	0.245 (Large)
Accel Max (m/s-2)	2.93 ± 0.42	3.37 ± 0.30	2.98 ± 0.58	3.21 ± 0.15	3.21 ± 0.24	2.17	0.081	-	0.110 (Moderate)
Decel Max (m/s-2)	-2.81 ± 0.38	-3.46 ± 0.20	-2.80 ± 0.43	-2.79 ± 0.21	-3.02 ± 0.24	6.34	0.000	L - LH, MB, RH	0.266 (Large)
Max Speed(m/s-1)	17.31 ± 1.58	18.32 ± 1.09	16.16 ± 2.20	18.15 ± 0.95	17.33 ± 1.43	3.91	0.006	MB - L, RH	0.183 (Large)
Acc/ Dec (m/s-2)	-1.05 ± 0.11	-0.98 ± 0.13	-1.07 ± 0.11	-1.15 ± 0.05	-1.07 ± 0.10	3.10	0.021	-	0.150 (Large)
Accum Acce Load (A.U)	485.57 ± 91.54	402.22 ± 92.13	365.79 ± 63.41	530.92 ± 88.82	528.27 ± 103.72	12.62	0.000	MB - LH, RH, S; L - RH, S	0.419 (Large)
Acute Mean Load (RA)	861.12 ± 358.49	784.35 ± 331.57	737.11 ± 303.17	798.83 ± 440.69	933.5 ± 288.60	1.04	0.392	-	0.039 (Small)
Chronic Mean Load (RA)	774.80 ± 146.87	684.29 ± 212.04	666.34 ± 137.91	732.16 ± 202.10	770.03 ± 124.45	2.38	0.057	-	0.085 (Moderate)
AC Ratio (RA)	1.07 ± 0.35	1.20 ± 0.53	1.07 ± 0.35	1.00 ± 0.45	1.19 ± 0.32	0.758	0.555	-	0.029 (Small)

LH: Left Hitter, RH: Right Hitter, MB: Middle Blocker, S: Setter and L: Libero

* Examples for Source of difference column: (A) – (B) denotes significant differences between A and B; (A)-(B)-(C) denotes significant differences for all possible pairwise combinations for A, B and C. (A)-(B),(C) denotes significant differences between A and B, and also between A and C, (A),(B) – (C) denotes significant differences between A and C, and also between B and C
